# Research Progress on Recycling Technology of Spent Lithium-Ion Battery Electrodes: From the Perspective of Physical Separation and Intelligent Recognition

**DOI:** 10.3390/molecules31173040

**Published:** 2026-08-29

**Authors:** Yongjie Bu, Chengbo Yu, Xingxing Deng, Hongliang Zhang, Ruibo Ma

**Affiliations:** 1 School of Resources, Environment and Safety Engineering, Hunan University of Science and Technology, Xiangtan 411201, China; 25020101101@mail.hnust.edu.cn (C.Y.); mrb7772026@outlook.com (R.M.); 2 Key Laboratory of Sichuan Province for Lithium Resources Comprehensive Utilization and New Lithium Based Materials for Advanced Battery Technology, Chengdu 610041, China; 3 School of Minerals Processing and Bioengineering, Central South University, Changsha 410083, China; hongliang.zhang@csu.edu.cn

**Keywords:** spent LIBs, electrode recycling, physical separation, sensor-based sorting, intelligent sorting

## Abstract

Against the global drive for energy conservation and emission reduction, lithium-ion batteries (LIBs) have witnessed increasingly widespread adoption across diverse sectors, thereby generating an urgent imperative for the efficient recycling of electrodes from spent LIBs. This paper presents a comprehensive review of recent research progress in this domain. It begins by elucidating the fundamental architecture of LIBs and the key physicochemical characteristics of their electrode materials, followed by a critical analysis of the major technical bottlenecks inherent in current recycling processes. While conventional separation techniques are briefly discussed, the primary emphasis is placed on reviewing deep learning-based visual inspection systems, with particular attention to YOLOv8-based approaches reported in the literature. Finally, future development trajectories are delineated, and pivotal research directions including the optimization of multi-technology integration and the expansion of training dataset scale and diversity are proposed to facilitate the large-scale, standardized industrial implementation of electrode recycling technologies.

## 1. Introduction

Amid the global focus on energy conservation and emission reduction, the energy demand paradigm has undergone a transition toward advanced energy storage systems [1]. In this context, the lithium-ion battery (LIB) industry has experienced substantial growth in recent years. Presently, the lithium-ion battery market is classified according to the cathode materials employed, encompassing lithium cobalt oxide (LCO), lithium manganese oxide (LMO), nickel-cobalt-manganese ternary materials (NCMs), and lithium iron phosphate (LFP) [2]. Among these, LFP warrants particular attention in the recycling literature because its recycling differs fundamentally from that of Ni- and Co-containing oxide cathodes: the lower intrinsic value of LFP and the absence of cobalt and nickel mean that its economic viability depends on efficient physical liberation and lithium recovery rather than on the recovery of high-value transition metals [3,4,5]. LFP-specific separation routes therefore receive particular emphasis throughout this review [6].

With the global advancement of electrification, energy security and environmental sustainability issues have grown increasingly prominent [4]. Consequently, when deliberating the long-term development of lithium-ion batteries (LIBs) in the power and energy storage sectors, it is imperative to conduct research on the fabrication of higher-performance products while attaching greater significance to recycling technologies [7].

**Figure 1:**
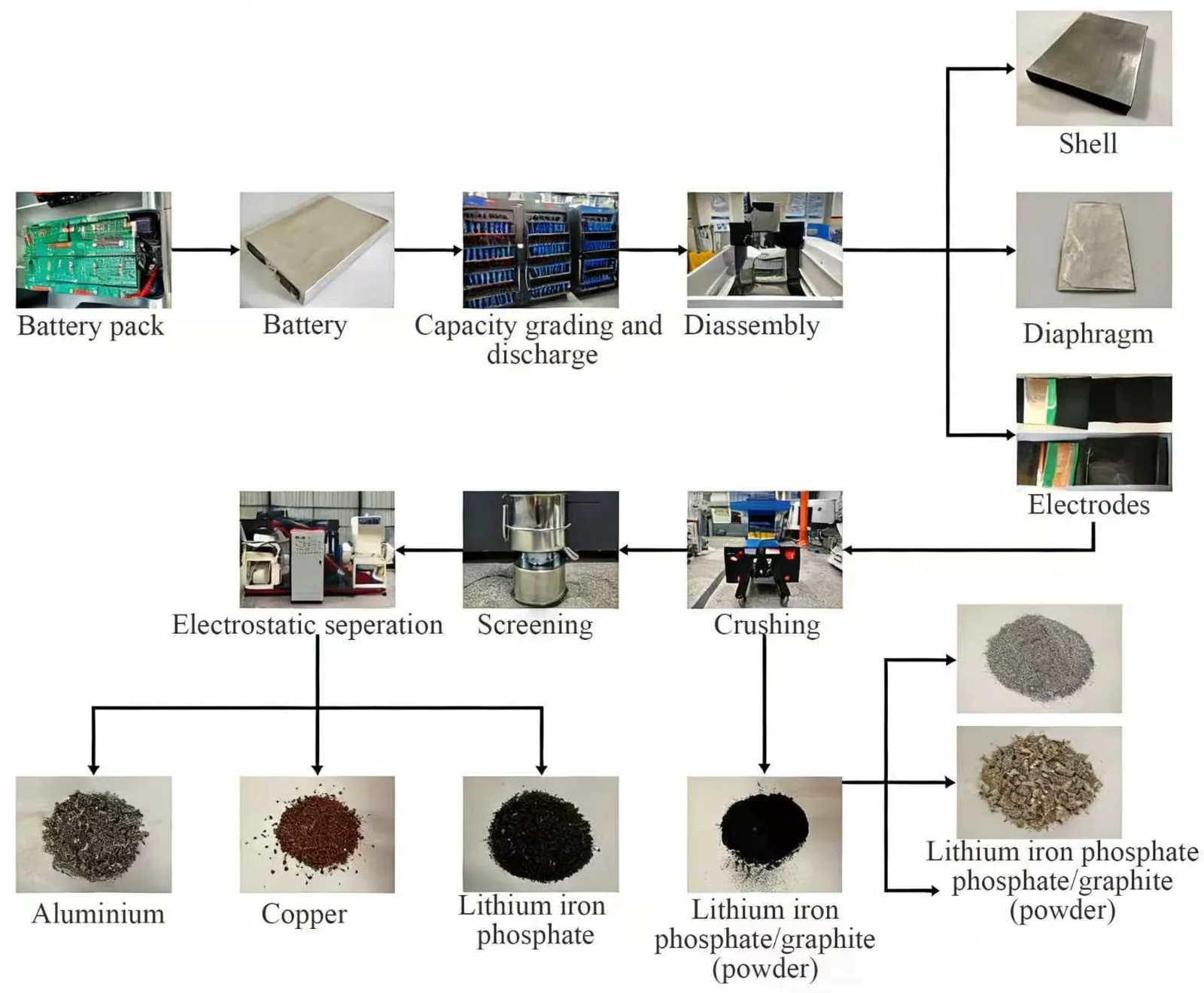
Recovery process of a waste lithium iron phosphate battery [9].

A lithium-ion battery is a multi-component assembly whose cathode—a lithium transition-metal oxide (LCO or NCM) or the olivine phosphate LiFePO_4_ (LFP)—is coated on an aluminum current collector, while the anode is coated on a copper current collector [6,7]. The electrodes are fixed by polymeric binders and enclosed with an electrolyte, separator, and casing. For recycling, the separation strategy is governed by electrode structure and binder chemistry rather than by any single universal rule: the graphite anode is generally bound more weakly than the cathode coating and is correspondingly easier to liberate; the cathode coating, bonded to the aluminum foil by the PVDF binder, adheres strongly and resists mechanical delamination [8]; and the cells contain both valuable metals (Li, Ni, Co, Mn, Cu, Al) and hazardous components that must be recovered and contained, respectively [6,7], as illustrated in Figure 1.

**Figure 2:**
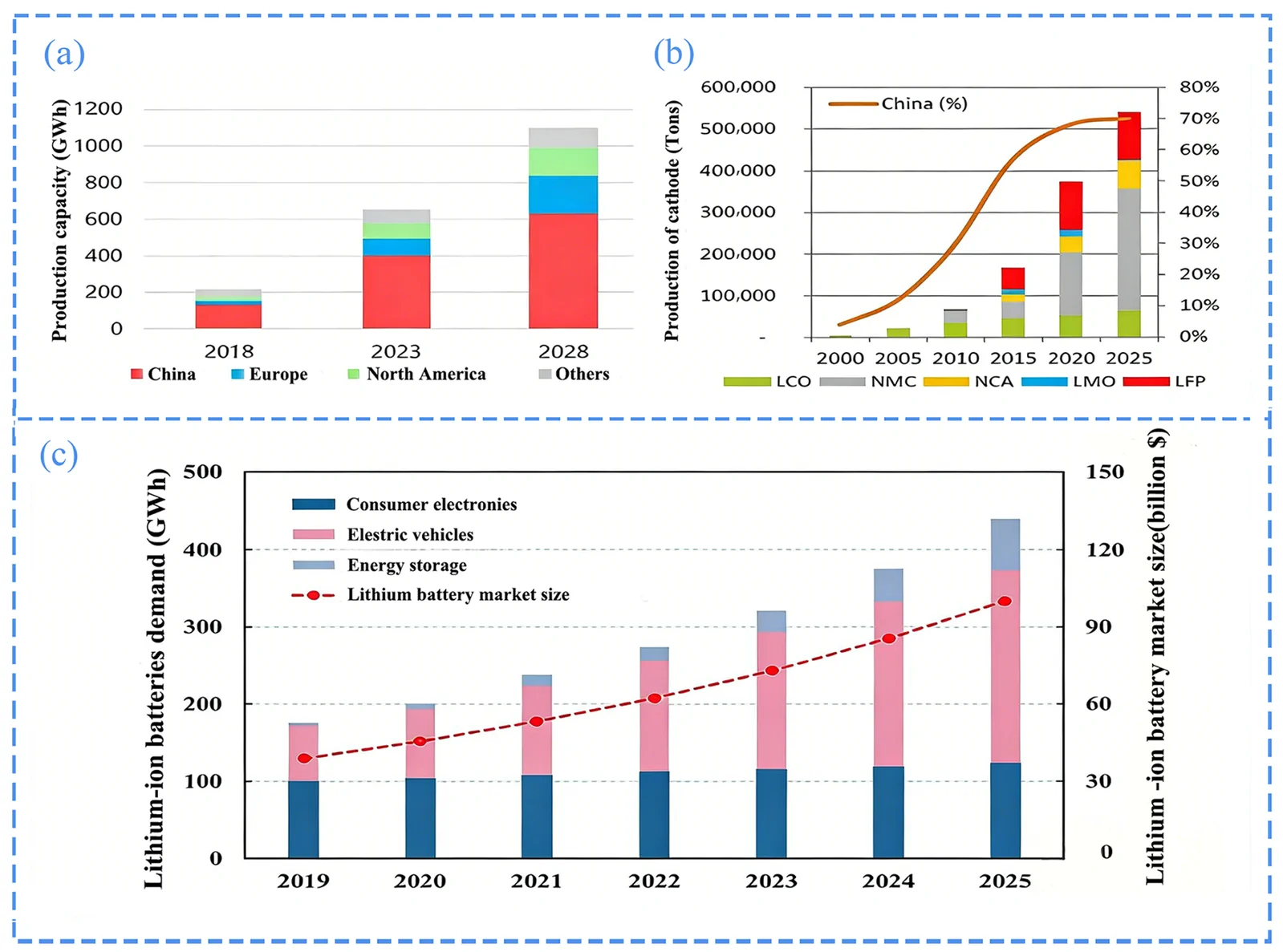
(**a**) Prediction of LIBs production capacity by region, (**b**) Cathode production trend of different types of LIBs [19], (**c**) LIBs market size and demand; Source [20].

The lithium-ion battery industry boasts broad prospects and serves as a key sector for driving global low-carbon transition and achieving sustainable development. It is indispensable in fields such as energy storage and electric vehicles. However, the industry’s development is also confronted with numerous problems and challenges: On one hand, the diversity of lithium-ion batteries makes it difficult to standardize industrial recycling processes. In particular, lithium iron phosphate batteries have relatively low recycling value, resulting in insufficient motivation for the research and development of recycling technologies [5]. On the other hand, existing recycling technologies have inherent drawbacks:

Pyrometallurgical processes consume high energy, tend to emit polluting gases, and waste lithium resources [10]; hydrometallurgical processes have long workflows, high wastewater treatment costs, and may use toxic reagents; most direct regeneration methods are still in the laboratory stage; physical separation methods require the prior removal of organic binders, and traditional removal methods are likely to damage the integrity of battery materials [11]. If waste lithium-ion batteries are improperly handled, they pose risks such as leakage, combustion, and explosion. The fluorides they contain may damage the ozone layer; heavy metals like copper and iron, as well as organic solvents, can contaminate soil and water sources [12]. Improper handling of spent batteries poses well-documented environmental hazards, including the release of fluorides, heavy metals and flammable organic solvents [13]; the combustion or decomposition of organic binders, separators, and other components will produce toxic substances such as carbon monoxide and aldehydes, endangering the ecological environment and human health [14]. Figure 2 consists of three subgraphs, illustrating the global production, demand and supply-demand contradiction of lithium resources for lithium-ion batteries, especially lithium iron phosphate batteries (LFPBs), thus providing data support for the necessity of battery recycling [15].

This review focuses specifically on physical separation and sensor-based intelligent recognition as the pre-chemical stages of the LIB recycling chain. Downstream processes including hydrometallurgical leaching, electrochemical electro-deposition of metals, membrane concentration, and solvent extraction are essential complementary stages but fall outside the scope of this review; readers are directed to authoritative reviews on these topics [16,17]. Industrial metallurgical routes downstream of mechanical processing have been critically reviewed by Brückner et al. [18].

The distinctive contribution of this review, relative to existing surveys of lithium-ion battery recycling, lies in three elements. First, whereas most prior reviews catalogue separation technologies in isolation, we adopt a two-dimensional analytical framework that classifies every technology by its physical principle on one axis and by its intelligence level (rule-based conventional operation versus data-driven deep-learning operation) on the other; this framework is formalized in Table 3 and applied consistently throughout Section 3 and Section 4, so that technologies are compared along both dimensions rather than described independently. Second, we deliberately confine the scope to the pre-chemical physical and sensor-based stages of the recycling chain—a boundary that is frequently blurred in prior reviews but is essential for meaningful technology benchmarking. Third, we differentiate the maturity of each technology through a transparent technology readiness level (TRL) assessment and through a task-level taxonomy that separates studies conducted directly on crushed electrode materials from those conducted on adjacent tasks such as e-waste classification, battery-level detection, and manufacturing defect inspection. By holding these distinctions fixed, the review moves beyond a descriptive compilation toward a structured, critical comparison of what is industrially proven and what remains at the laboratory frontier.

It must be emphasized that spent LIBs cannot be simply landfilled or incinerated. Their components—fluoride-containing electrolytes, heavy metals (Co, Ni, Mn, Cu), flammable organic solvents, and fluorinated polymer binders—pose serious environmental and human health risks if improperly disposed of. The core purpose of physical separation and intelligent sorting, as reviewed herein, is to transform these hazardous wastes into purified, marketable secondary material streams that re-enter the manufacturing supply chain, thereby realizing both resource circularity and environmental protection.

To ensure transparency and systematic coverage, this review followed a structured literature search strategy. The databases searched included Web of Science, Scopus, Google Scholar, and CNKI, using keywords such as “spent lithium-ion battery”, “electrode recycling”, “physical separation”, “eddy current”, “XRT sorting”, “deep learning”, and “YOLO”. The time range covered 2015–2025, with a few seminal older references. Only peer-reviewed journal articles, conference proceedings, and authoritative industrial reports were included. Patents and purely theoretical studies without experimental validation were excluded. This methodology allows the review to be reproducible and avoids selective citation.

All retrieved records were screened in three stages: (i) duplicate removal and title screening, (ii) abstract screening against the inclusion criteria, and (iii) full-text assessment. Studies were retained only if they reported primary experimental data, industrial-scale demonstrations, or methodological advances directly relevant to the physical separation or sensor-based intelligent recognition of battery electrode materials; review articles were used for contextual framing rather than as the basis for quantitative conclusions, and preference was given to primary experimental and industrial reports wherever overlapping results existed [21,22,23,24]. The search was not geographically restricted, and the review deliberately integrates contributions from European research groups, including those at TU Braunschweig [25], KU Leuven [26], and the Helmholtz Institute Freiberg [27], alongside work from Asia and North America, to provide a balanced international perspective.

## 2. Fundamental Characteristics of Spent Lithium-Ion Battery Electrodes

### 2.1. Structure of Lithium-Ion Batteries

**Figure 3:**
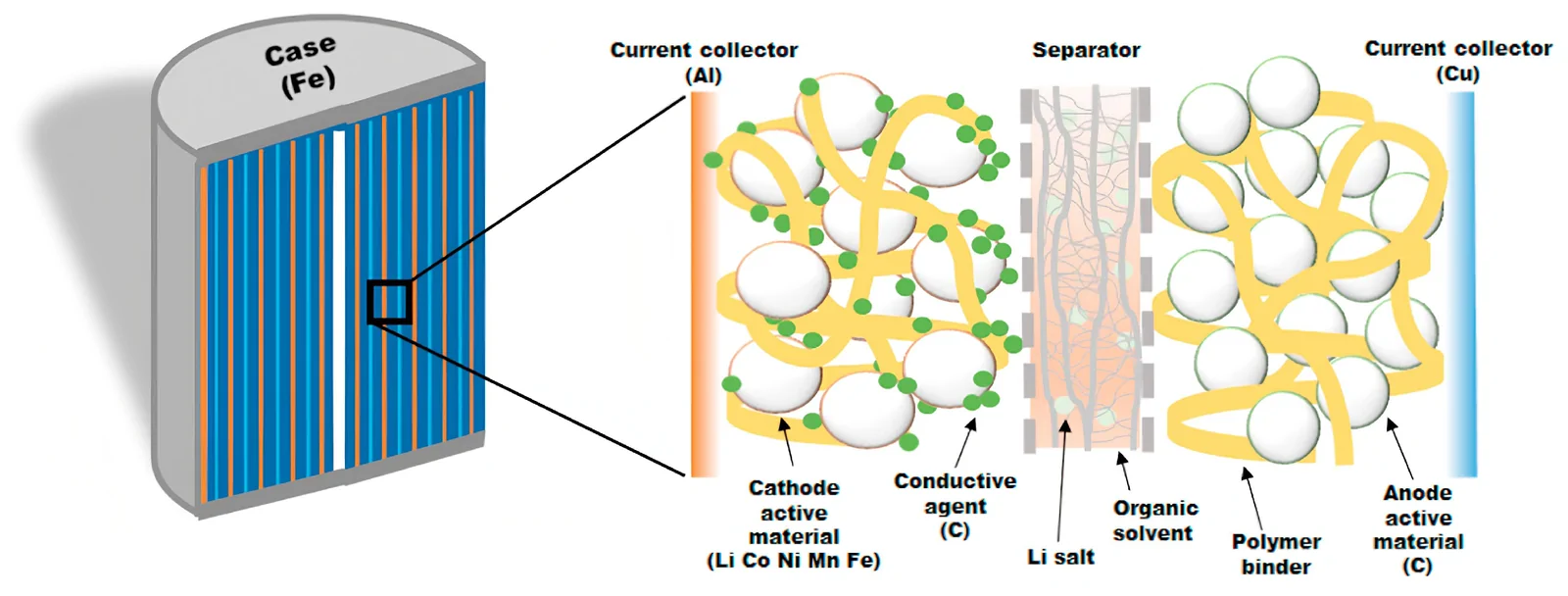
A schematic diagram of the LIB components and their constituent elements at issue [21].

In the fundamental structure of a lithium-ion battery, the cathode and anode assemblies are separated by a porous separator impregnated with a lithium salt electrolyte. The cathode, supported by an aluminum current collector, is composed of lithium transition metal oxide (Li\Co\Mn\Fe) active materials, conductive carbon, and a polymer binder, enabling the reversible deintercalation of lithium ions during charge–discharge cycles. The anode, anchored to a copper current collector, accommodates lithium-ion intercalation via carbon-based active materials. While physically isolating the electrodes, the separator permits the free migration of lithium ions through the electrolyte, which is critical for ensuring battery safety [28]. This layered structural design underpins the battery’s capacity, rate capability, and cycle life, forming the core foundation of modern electrochemical energy storage technology, as illustrated in Figure 3.

Table 1 shows the main components of lithium cobalt oxide battery, lithium nickel manganese cobalt oxide (NMC) battery, and lithium iron phosphate (LFP) battery, where the cathode material accounts for 41 wt%, 26 wt%, and 25 wt% of the battery, respectively. Recycling these spent cathode materials can not only recycle lithium but also recover nickel, cobalt, manganese, and aluminum.

### 2.2. Electrode Recycling Methods

This review focuses on the first two stages of the spent lithium-ion battery electrode recycling chain: the pretreatment stage, which includes discharging, dismantling, crushing, and binder removal, and the physical separation and intelligent sorting stage, which encompasses eddy current separation, electrostatic separation, magnetic separation, pneumatic separation, color sorting, X-ray transmission sorting, deep learning-based recognition, and multi-sensor fusion. The technical principles, research progress, and industrial applicability of these physical unit operations are systematically reviewed and critically assessed [30].

The pretreatment stage has been comprehensively reviewed by Kim et al. [21]. The present review therefore focuses on the subsequent separation stages, where physical and intelligent sorting technologies are applied to the crushed electrode material stream [31].

The copper current collector serves as the anode substrate, operating under reducing potentials where copper is thermodynamically stable. Its high density relative to graphite anode material facilitates gravity-based separation after crushing. However, copper foil is prone to brittle fracture during mechanical processing, generating fine metallic particulates that can contaminate the cathode active material stream and cause short-circuits if residual Cu persists in regenerated cathode materials [32]. These physical characteristics make copper recovery primarily a problem of (i) preventing over-grinding during crushing to preserve foil integrity, and (ii) employing density- and conductivity-based sorting methods to achieve high-purity separation from graphite and other low-density components. The full recycling chain, from discharge and dismantling through crushing to physical separation, has been described at the process level by Werner et al. [33].

#### 2.2.1. Research Background on Electrode Foil Classification

Copper and aluminum foils serve as current collectors in lithium-ion batteries, with distinct roles determined by the electrochemical environment of each electrode. The cathode aluminum foil operates under oxidizing potentials, where aluminum forms a stable passivation layer that prevents further corrosion [34]. The anode copper foil operates under reducing potentials, where copper is thermodynamically stable. These complementary electrochemical requirements make the choice of current collector material fundamentally constrained by the electrode’s operating potential window. A central challenge in electrode recycling is the strong adhesion between the active material coating and the current collector foil. This adhesion is primarily provided by polyvinylidene fluoride (PVDF) binder, which forms mechanical interlocking and hydrogen bonds with both the active material particles and the foil surface [35]. The separation of cathode materials from aluminum foil is particularly challenging because: (i) the PVDF binder creates a robust interface that resists mechanical delamination; (ii) aluminum foil is ductile and prone to fragmentation during crushing, generating fine metallic impurities that contaminate the active material stream; and (iii) the similar density of Al foil and cathode materials limits the effectiveness of density-based separation. Conversely, copper foil is more readily separated from graphite anode material by gravity-based methods. The distinct separation behaviors of anode and cathode assemblies necessitate different physical separation strategies, as systematically compared in the following sections.

#### 2.2.2. Anode Materials and Current Collector

From a recycling perspective, the anode assembly is generally the more tractable side of the cell for two reasons: the graphite active material is bonded to the copper foil by water-based binders (typically carboxymethyl cellulose/styrene-butadiene rubber) rather than the PVDF binder used on the cathode [7,36], and copper is markedly denser and more electrically conductive than graphite. These properties allow the graphite coating to be liberated by comparatively mild mechanical or thermal treatment and the copper fragments to be recovered by gravity-, eddy-current-, or electrostatic-based sorting [33,37]. Anode recovery is nonetheless influenced by binder chemistry and electrode ageing, which alter interfacial adhesion and liberation behaviour and must be optimized for aged rather than pristine electrodes [7,38]. Recent work has moved beyond treating the anode as low-value tailings toward the co-recovery of copper and regenerable graphite: Kong et al. [39] reported an integrated route in which the recovered graphite was upgraded into an environmental catalyst, and attrition-assisted flotation has been shown to improve the separation of graphite from copper-bearing fractions [40,41,42]. Two further anode-detachment routes have been demonstrated experimentally: electrical pulsed discharge delaminated over 95% of the graphite from the copper foil while preserving its crystallinity, after which wet sieving, elutriation, and acid leaching removed iron and copper contaminants [43]; and simple water immersion of PVDF-bound anodes from end-of-life cells triggered delamination through the reaction of remnant lithiated graphite with water, with a subsequent 500 °C anneal regenerating graphite comparable to commercial material [44]. Systematic flotation studies further confirm that graphite is strongly hydrophobic whereas the metal-oxide cathodes are largely hydrophilic, so flotation separation is chemistry-dependent rather than universal [45], and roasting- and pyrolysis-assisted flotation removes the surface organic layer to sharpen this wettability contrast [46]. The principal remaining bottleneck is therefore not material recognition but liberation quality—avoiding over-grinding that embrittles the copper foil into fine particulates contaminating the cathode stream, and achieving complete graphite detachment so that both fractions meet market purity requirements [38].

#### 2.2.3. Cathode Current Collector Recycling

The aluminum current collector is the principal metallic component of the cathode assembly. In contrast to the anode, cathode separation is difficult not because aluminum is hard to identify, but because the active-material coating is strongly bonded to the foil by the PVDF binder—which forms mechanical interlocking and hydrogen bonds with both the particles and the foil surface —while the ductile foil fragments during crushing and contaminates the active-material stream [8]. Binder removal is therefore a prerequisite for high-purity aluminum recovery, and the appropriate method is chemistry-specific: for LFP cathodes, thermal pyrolysis in the approximate range of 500–650 °C decomposes the binder and detaches the coating while preserving the olivine LiFePO_4_ phase [47,48]; for NCM and LCO cathodes, solvent-based dissolution and lower-temperature thermal treatment have been investigated [49]. Complementary experimental routes include controlled pyrolysis of mixed-type batteries, which recovered the aluminum foil at 99.4–99.9% purity; gradient pyrolysis, which separates the foil at 350 °C and removes organic impurities at 650 °C [50]; and direct reuse of the recovered aluminum and copper current collectors in new cells without re-refining [51]. These examples indicate that, on the cathode side, pretreatment—rather than recognition during sorting—is the rate-limiting stage, and that the optimal binder-removal route depends on the cathode chemistry rather than being universal.

**Figure 4:**
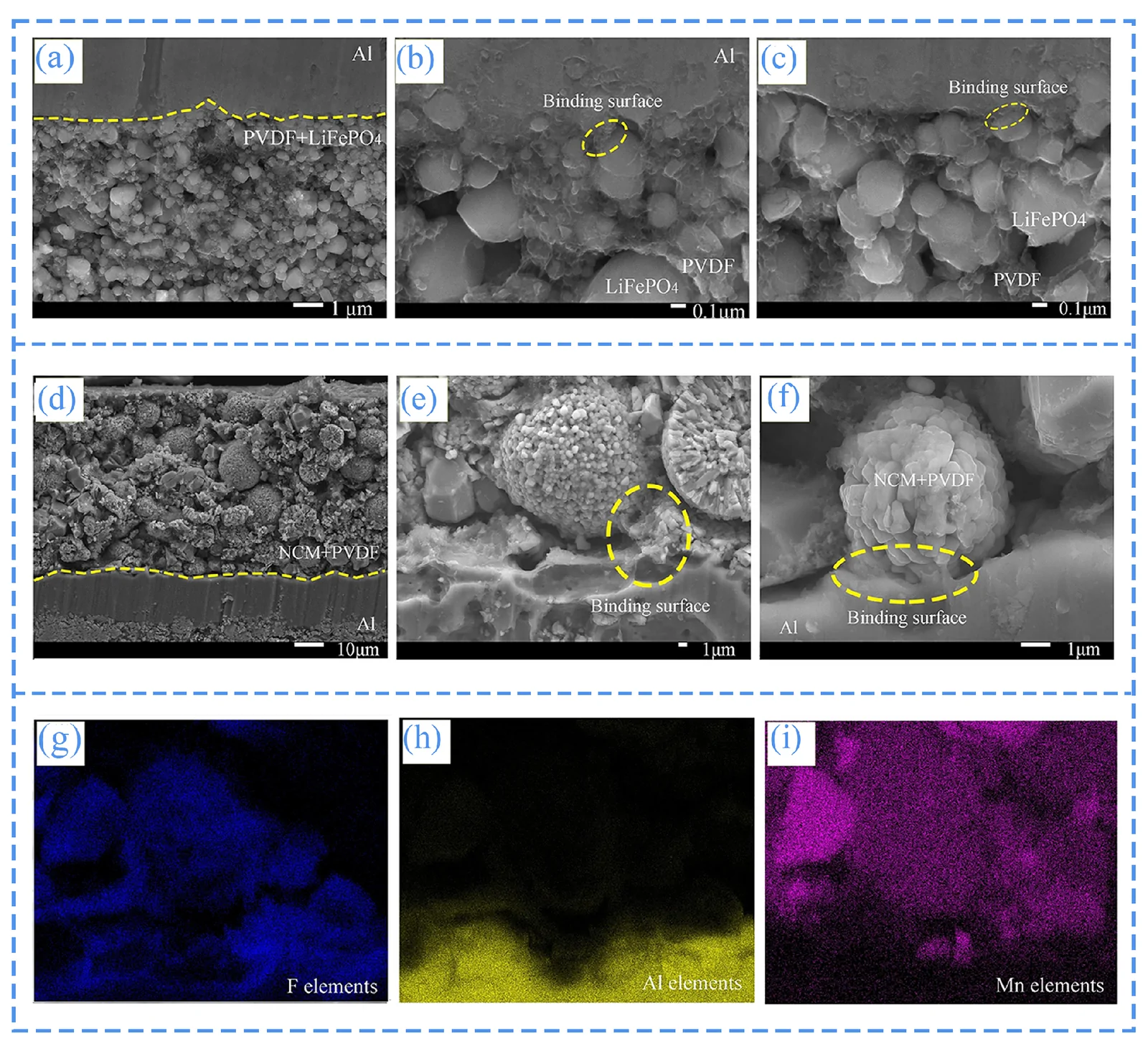
(**a**–**c**) SEM analysis of binding surfaces in LFP plates; (**d**–**f**) SEM analysis of binding surfaces in NCM plates [8]; (**g**–**i**) EDS analysis of binding surfaces in NCM batteries [52].

In the examined cathodes, Al is confined to the current collector, while NCM and PVDF correspond to their respective characteristic elements; no chemical interpenetration of elements was observed at the interface, where only physical adhesion was detected [8]. Figure 4 reveals the interfacial adhesion morphology and element distribution characteristics of the cathode material–PVDF–aluminum foil structure.

### 2.3. Chapter Summary

This chapter systematically describes the fundamental characteristics of spent LIB electrodes and introduces a two-dimensional classification framework for electrode recycling technologies. The framework categorizes separation methods by physical principle on one axis and intelligence level on the other. The layered structure of LIBs is presented with mass fraction data for different cathode chemistries. The recycling value and technical challenges of anode (Cu) and cathode (Al) current collectors are discussed separately, with a critical comparison of their distinct separation behaviors. SEM/EDS images reveal the interfacial adhesion morphology between electrode materials and current collectors—a key factor governing physical separation efficiency. In summary, the physicochemical properties of electrode materials and their bonding state with current collectors form the basis for selecting and optimizing downstream sorting and recovery strategies, and the proposed framework provides a systematic basis for comparing technologies across Section 3 and Section 4.

## 3. Conventional Physical Separation Technologies for Electrodes

### 3.1. Eddy Current Sorting

Eddy current separation (ECS) is a non-contact physical method that exploits differences in electrical conductivity (σ) and density (ρ) among particulate materials. The technology is particularly suitable for separating non-ferrous metallic particles (Cu, Al) from non-conductive materials in crushed spent LIB streams [53].

Two main ECS configurations are employed: the linear motor type and the alternating permanent magnet (APM) type. In the APM configuration—more common in recycling applications—a rotor with alternating-polarity permanent magnets spins at high speed beneath a conveyor belt. When electrically conductive particles enter the alternating magnetic field, the time-varying magnetic flux induces eddy currents within the particles. These eddy currents, in turn, generate a secondary magnetic field that opposes the primary field, producing a repulsive Lorentz force. The magnitude of this repulsive force is proportional to the σ/ρ ratio of each particle, causing particles with different conductivity-to-density ratios to follow distinct trajectories upon leaving the conveyor belt [54].

For spent LIB electrode recycling, ECS has demonstrated effective separation of aluminum foil fragments from copper foil fragments. However, the technology faces three key limitations in this application context: (1) particle size sensitivity—ECS efficiency drops sharply for particles below ~2 mm because the induced eddy current magnitude scales with particle cross-sectional area; (2) shape dependency—flaky electrode fragments generate unpredictable Lorentz force distributions due to variable orientation in the magnetic field [55]; and (3) multi-material interference—the presence of partially delaminated electrode fragments produces intermediate σ/ρ values that reduce separation sharpness. These limitations motivate the integration of complementary separation methods and intelligent sorting technologies to achieve industrial-grade separation purity [37].

**Figure 5:**
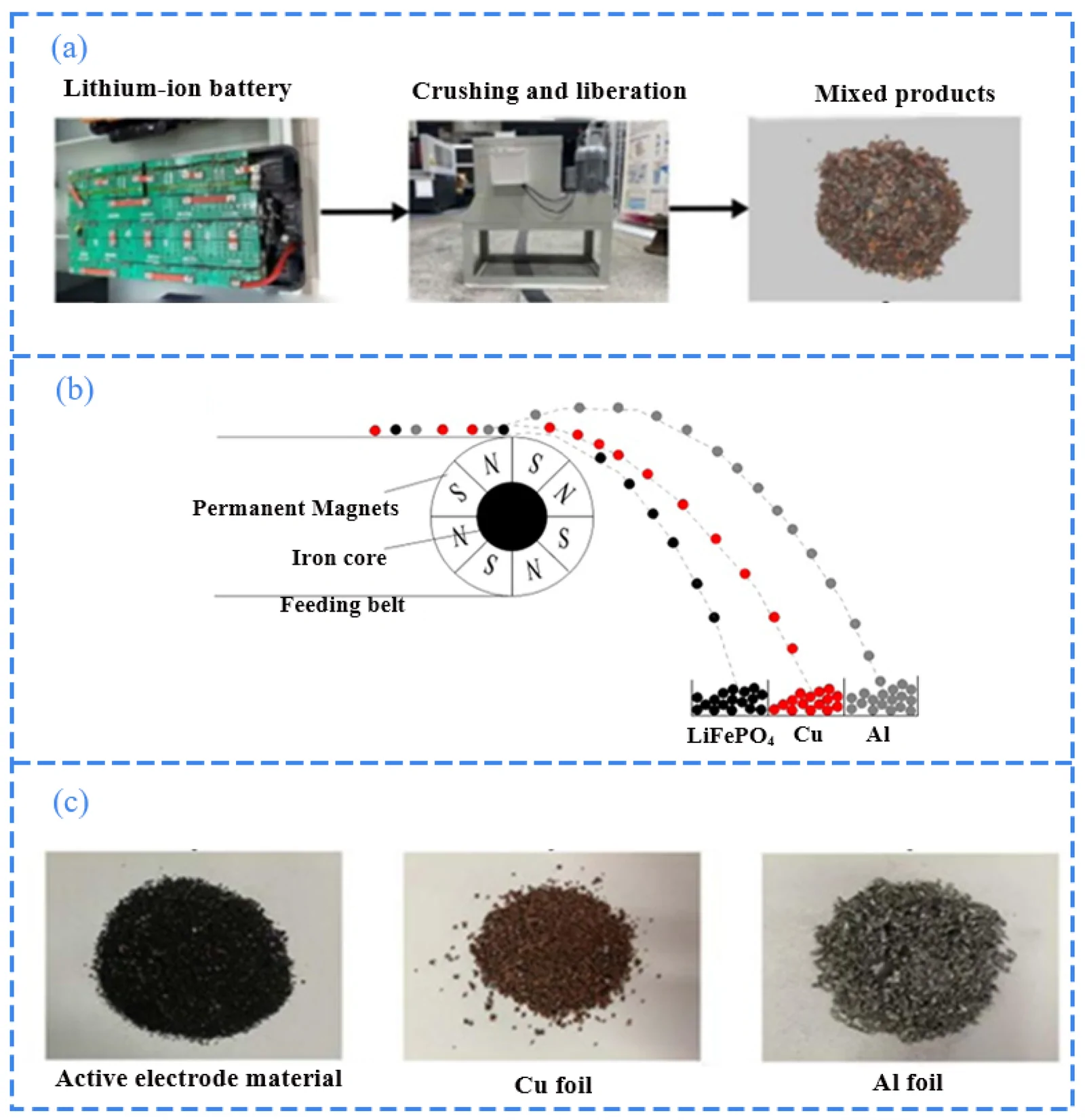
(**a**) Battery crushing and liberation. (**b**) The eddy current separation used for the recycling of the LFP-based LIBs [53]. (**c**) Separated products [54].

The separation of spent lithium-ion battery components was achieved using two complementary physical separation techniques, as illustrated in Figure 5. In the present section, we focus exclusively on eddy current separation. In the complete separation circuit of Figure 5, a permanent magnetic drum separator was first employed to remove ferromagnetic impurities, while the weakly paramagnetic LiFePO_4_ cathode material was partially concentrated via high-gradient magnetic separation (HGMS) onto the rotating drum. In contrast, the non-magnetic metallic current collectors—copper and aluminum foils—exhibit negligible magnetic response and thus pass through the magnetic separation stage unaffected. These non-ferrous metal fractions are subsequently separated in the eddy current separator, where the induced repulsive Lorentz force deflects the highly conductive Cu and Al particles along distinct trajectories based on their differing conductivity-to-density ratios, enabling their selective collection [56].

The majority of ECS studies on LIB materials have been conducted using clean, pre-sorted Cu and Al foil fragments under controlled laboratory conditions [53,54,56]. Studies employing realistic post-crushing electrode streams—which contain partially delaminated fragments, binder residues, and variable particle morphologies—are scarce. The drop in separation efficiency below ~2 mm particle size [55] is particularly concerning for industrial application, as mechanical crushing typically generates a substantial fine fraction. Recent work by Erdogan et al. [57] provides the first systematic evaluation of ECS performance on realistic crushed LIB streams, reporting efficiency reductions of 15–25% compared to idealized particle mixtures, primarily due to shape-irregularity effects and partial delamination. Validation of ECS on unsorted, multi-chemistry crushed electrode streams with quantified purity and recovery metrics remains an active research need. Bai et al. [58] advanced this by developing an eddy current separation motion model that explicitly accounts for the particle shape coefficient and air resistance; combined with ball-milling pretreatment, their experiments recovered 95.9% Cu, 97.1% Al, and 93.4% LiFePO_4_ at a rotor speed of 1000 rpm and a shape coefficient of 1.5, demonstrating that particle shape—rather than conductivity contrast alone—governs ECS trajectory and recovery.

### 3.2. Electrostatic Sorting

Electrostatic separation exploits differences in surface electrical conductivity of materials. In a typical corona-electrostatic separator, particles are fed onto a rotating grounded drum and exposed to a high-voltage corona discharge. Conductive particles lose their charge quickly to the drum and are ejected by centrifugal force, while non-conductive particles retain their surface charge and stick to the drum, later being brushed off [56].

In the context of spent LIB recycling, electrostatic sorting has been applied to separate conductive metal foils (Cu, Al) from non-conductive components such as polymeric separators and organic binders after crushing [54]. It is particularly effective for fine particles in the range of 0.1–5 mm [56]. Silveira et al. [59] obtained a conductive fraction containing 98.98% metals and a nonconductive fraction with 99.6% polymers by corona-roll electrostatic separation, and a combined electrostatic–magnetic sequence recovered over 98% of NMC, LMO/NCA, and LFP electrodes from coarsely shredded cells [60]. However, its performance is sensitive to humidity, requiring pre-drying of the feed material, and it may be affected by the irregular shapes of crushed electrode fragments. Despite these limitations, electrostatic sorting remains a low-energy, chemical-free method that can be integrated as a polishing step after pneumatic or eddy current pre-separation.

### 3.3. Magnetic Separation

Magnetic separation exploits differences in magnetic susceptibility among particulate materials. In the context of spent LIB electrode recycling, its applicability is limited but non-negligible.

LiFePO_4_ is weakly paramagnetic, with a mass magnetic susceptibility on the order of 10^−6^ m^3^/kg. Conventional permanent magnetic drum separators, which typically generate field strengths of 0.1–0.5 T at the drum surface with limited field gradients, are generally insufficient to capture such weakly responsive particles. In contrast, high-gradient magnetic separation (HGMS) employing ferromagnetic matrix elements can induce local field gradients of 10^3^–10^4^ T/m, which significantly enhances the magnetic force on fine LFP particles and enables their effective capture despite their weak intrinsic magnetism. Bi et al. [53] demonstrated LFP separation using a magnetic separator; the effective mechanism likely involves HGMS amplification rather than direct magnetic attraction by a permanent magnet drum. Additionally, partial oxidation of Fe^2+^ to Fe^3+^ during thermal pretreatment may slightly enhance the effective magnetic moment of LFP particles.

Ferromagnetic impurities—including metallic iron from casing wear, steel fragments from crushing equipment, and certain NMC cathode variants with higher transition metal content—are more readily separable by conventional low-intensity magnetic separators. These impurities represent a minor fraction of the electrode stream but must be removed to prevent contamination of downstream processes.

Magnetic separation plays a supplementary role in electrode recycling, primarily for tramp iron removal and, potentially, for LFP-specific HGMS-based sorting. No peer-reviewed study has demonstrated industrial-scale (TRL ≥ 6) magnetic separation of LFP from crushed electrode streams under realistic throughput conditions. The cited laboratory studies employed idealized feed materials and small batch sizes [53]. More recent experiments demonstrate greater promise: high-intensity magnetic separation recovered LiFePO_4_ from graphite based on their contrasting magnetic susceptibilities [61]; high-gradient magnetic separation captured approximately 99% of LFP particles using steel-matrix field intensification; and magnetic separation coupled with low-temperature pyrolysis at 400 °C achieved 97.2% LFP recovery at 99.1% purity [62]. Validation at pilot scale with realistic, multi-chemistry electrode feedstocks is required before magnetic separation can be considered an industrially viable component of the electrode recycling toolkit.

### 3.4. Pneumatic Sorting

**Figure 6:**
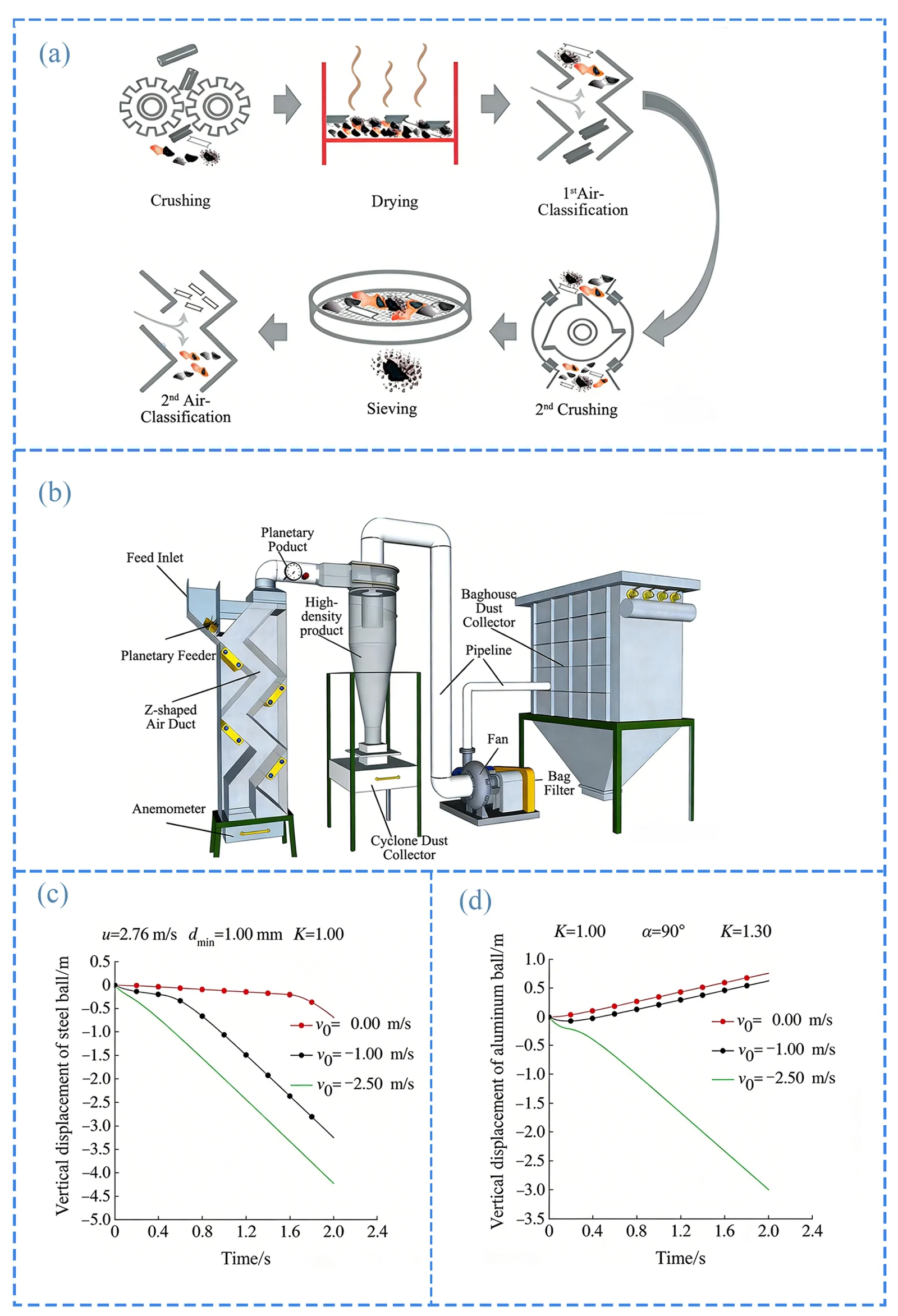
(**a**) The process flow diagram of the mechanical separation based on the two-step crushing [25]. (**b**) Structure diagram of pneumatic separator [63]. (**c**) The relationship between the vertical displacement of copper balls and time at different initial velocities. (**d**) The relationship between the vertical displacement of aluminum balls and time at different initial velocities [55].

Pneumatic separation uses air as the working medium to separate particulate materials based on differences in density, particle size, and aerodynamic shape. Figure 6 reveals the fundamental principle is differential settling: under the combined action of upward or horizontal airflow and gravity, particles of different mass and shape experience distinct drag-to-weight ratios, leading to divergent trajectories that enable component separation [25,63].

In spent LIB recycling, pneumatic separation serves two distinct operational roles. In the pretreatment stage, low-velocity airflow selectively removes lightweight separator fragments and fine carbon/lithium dust, preventing these materials from contaminating subsequent high-value metal separation steps. This pretreatment step has been shown to reduce the organic content in the metal-rich fraction by 60–80% [25]. In the core separation stage, carefully controlled airflow separates copper from aluminum foils based on their density contrast: vertical pneumatic classifiers achieve separation efficiencies of 93–96% for flaky Cu and Al foil mixtures [55], while horizontal cross-flow classifiers report >95% recovery for spherical copper and aluminum particles [64]. Combined pneumatic–electrostatic circuits have demonstrated Cu recovery rates ≥90% and Al recovery rates ≥ 89% at industrial pilot scale [64].

Despite its operational simplicity and high throughput, pneumatic separation has intrinsic limitations. Separation sharpness degrades when particle shapes are irregular—a common occurrence after mechanical crushing of electrode assemblies, where flakes, needles, and agglomerates coexist. Furthermore, hygroscopic electrode components can cause particle agglomeration at elevated humidity, reducing aerodynamic discrimination. These limitations highlight the need for pre-drying and multi-stage classification, as well as the potential for sensor-based intelligent sorting to serve as a polishing step downstream of pneumatic pre-concentration [65].

In an industrial pilot study [38], spent lithium-cobalt-oxide battery scrap was crushed and air-separated using a zigzag classifier; a low airflow velocity of 1.7–2.8 m/s selectively removed plastic separator fragments and fine carbon/lithium dust as a pretreatment step, preventing their contamination of subsequent electrostatic separation. This work established the operational window for pneumatic pretreatment that is now standard in industrial circuits.

Pneumatic separation is the most industrially mature physical separation technology for LIB electrode recycling, with demonstrated throughputs of several tons per hour and pilot-scale validation [64]. However, separation performance is highly feed-dependent: irregular particle morphologies from mechanical crushing can reduce separation sharpness by 20–40%, and hygroscopic components cause agglomeration at humidity >60% RH [26]. Most published studies report separation efficiencies under optimized, single-chemistry conditions; performance on mixed-chemistry feeds typical of industrial recycling streams has not been systematically characterized. The combination of pneumatic pre-concentration with intelligent sensor-based sorting represents a promising pathway to address these limitations.

### 3.5. Comparative Analysis of Physical Separation Technologies

To provide a clear benchmarking of the discussed physical methods, Table 2 summarizes their working principles, separation efficiency, advantages, limitations, and technology readiness level (TRL).

The TRL values reported here follow the widely used nine-level scheme, in which TRL 7–8 denote demonstration at pilot or industrial scale in a relevant environment and TRL 9 denotes proven operation in full commercial deployment. For each technology, the assigned TRL reflects its most mature, industrially demonstrated application within electrode recycling rather than its performance on every conceivable feedstock. Accordingly, eddy current separation (TRL 9) and pneumatic separation (TRL 9) are assigned the highest levels on the basis of documented commercial throughputs and pilot validation; electrostatic separation (TRL 8) is a proven but feedstock-sensitive polishing step; color/optical sorting (TRL 8) is industrially deployed yet limited to surface-discriminable materials; and XRT sorting (TRL 7) remains at demonstration scale, constrained by capital cost and, for multi-energy variants, by detector count-rate limits [38]. These values are harmonized with the classification in Table 3.

From a portfolio perspective, no single physical method is sufficient for electrode separation. Eddy current and pneumatic separation offer the highest throughput and are best positioned as bulk pre-concentration steps, but both degrade for fine (<2 mm) and irregularly shaped fragments [68]. Electrostatic separation complements these by handling fine particles, yet its humidity sensitivity mandates pre-drying. Color sorting is the least material-selective, being limited to surface-discriminable chemistries, whereas XRT provides the most information-rich discrimination at the highest capital and safety cost [40]. These complementary trade-offs—throughput, selectivity, and information content—motivate the multi-stage circuits and the sensor-based intelligent polishing discussed in Section 4.

### 3.6. Chapter Summary

This chapter provides a critical comparison of conventional physical separation technologies for electrodes. The working principles, target materials, and separation efficiencies of eddy current, electrostatic, pneumatic, color, and XRT sorting are described. A new comparative table (Table 2) is presented, evaluating each technology across seven dimensions: target material, working principle, separation efficiency, advantages, limitations, energy/chemical use, and technology readiness level (TRL). Key findings: eddy current and pneumatic sorting are mature (TRL 9) and suitable for large-scale pre-treatment; electrostatic sorting works for fine particles but is humidity-sensitive; color sorting is fast but affected by surface contamination; XRT sorting detects internal features and is dust-insensitive, but has high capital cost. No single technology meets all requirements, and multi-technology combinations are the practical industrial pathway.

Table 3 contrasts conventional and DL-enabled sorting across five physical principles. Conventional modes operate on fixed thresholds at TRL 7–9, while DL-enabled modes remain at TRL 3–4, reflecting a substantial validation gap. Deep learning offers differential benefits: YOLO enhances optical sorting via morphology-aware recognition, while XRT and electrostatic sorting gain from learned spectral/waveform features. The two modes are complementary—conventional for high-throughput pre-concentration, DL-enabled for adaptive polishing of variable feedstock.

**Table 3:** Systematic classification of electrode recycling technologies by physical principle and intelligence level.

Physical Principle	Conventional Mode (Sensor-Driven)	DL-Enabled Mode (Data-Driven)	Conventional TRL	DL-Enabled TRL	Application Scenario Comparison
Magnetic Separation	Permanent/electromagnetic separation of ferromagnetic metals	Magnetic response signal + ML for material type classification	9	4	Conventional: coarse separation of steel shells; DL: distinguishing stainless steel from magnetic electrode materials
Electrostatic Separation	High-voltage electrostatic separation of conductors/semiconductors/non-conductors	Discharge waveform + CNN for surface state recognition	8	3	Conventional: separation of Cu and Al foils; DL: identifying surface oxidation/coating layer variations
Pneumatic/Air Classification	Airflow + vibration classification by density/particle size	Air pressure/particle trajectory + ML dynamic parameter adjustment	9	4	Conventional: separation of lightweight separators; DL: adapting to particle size fluctuations and optimizing airflow
Optical/Color Sorting	RGB threshold or color difference detection	YOLO/ResNet object detection and classification	8	4	Conventional: distinguishing LFP vs. NMC by color; DL: identifying electrode fragmentation/coating detachment
X-Ray Transmission (XRT)	Dual-energy XRT classification by atomic number	CNN fusion of XRT images + spectral features	7	3	Conventional: distinguishing Cu foil/Al foil/active layer; DL: recognizing LCO/NMC/LFP mixed systems

## 4. Intelligent Sorting Technologies

Classification Note: In this chapter, sensor-based technologies are discussed in their intelligent” modality—i.e., when sensor signals are processed by deep learning algorithms rather than by rule-based thresholding. The conventional operation of the same sensors is covered in Section 3 and Table 2. This two-tier classification—detection principle vs. intelligence level—enables systematic comparison across the full spectrum from purely physical to fully intelligent operation.

### 4.1. X-Ray Transmission (XRT) Sorting

Multi-energy XRT detector development represents significant instrumental progress [24,69], but all validation to date has been conducted on mineral processing and e-waste streams, not on spent LIB electrode particles. The key differentiator for electrode applications—the ability to discriminate between cathode chemistries based on subtle atomic number differences—has been hypothesized but not experimentally validated on realistic, binder-contaminated, variable-thickness electrode fragments. The multi-stage deep learning architecture of Koyanaka & Kobayashi [70] for battery detection in e-waste provides a proof of concept for XRT + DL integration, but adaptation to electrode-level sorting has not been demonstrated. Wu et al. [71] provide the most relevant preliminary data, reporting promising discrimination of NMC from Al and Cu in shredded battery waste, but LFP discrimination remains challenging due to its lower and less distinctive Z_eff.

**Figure 7:**
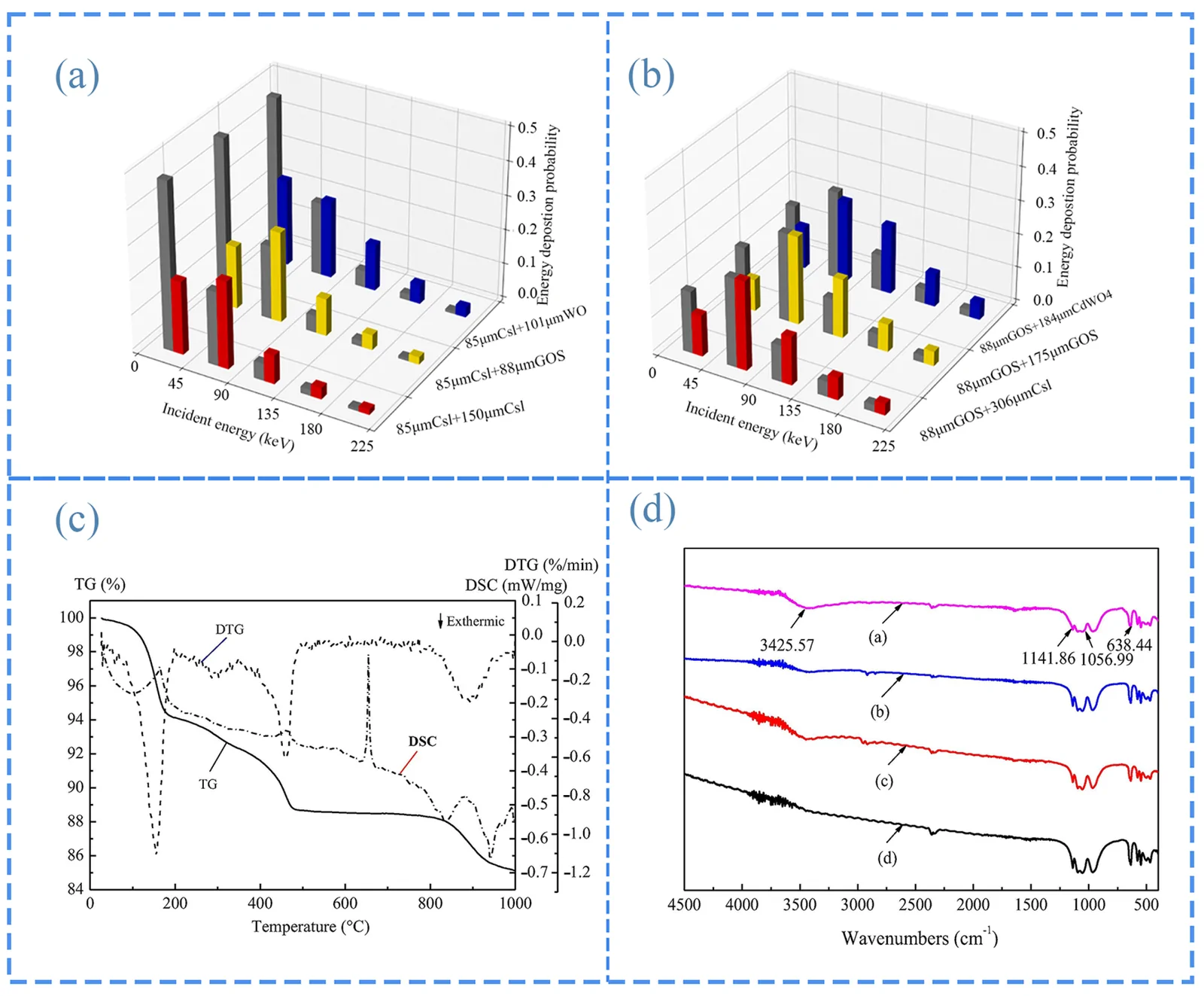
(**a**) Screening of the optimal scintillator material for the second layer of the five-layer detector. (**b**) Screening of the scintillator material for the third layer of the five-layer detector [24]. (**c**) TG-DSC curves of spent LIBs. (**d**) FTIR spectra of spent LiFePO_4_ batteries at different pyrolysis temperatures (a: Untreated; b: 450 °C; c: 500 °C; d: 550 °C) [47].

X-ray transmission imaging (XRT) is widely used for sorting materials [23]. However, conventional single-energy and dual-energy X-ray systems have poor ability to discriminate between materials with similar atomic number (Z), and the count rate of available multi-energy XRT detectors could not support high-speed industrial applications [24], as illustrated in Figure 7.

During the simulation design of the five-layer multi-energy X-ray imaging detector, a series of simulation analyses on the X-ray energy deposition distribution characteristics were conducted to determine the suitable scintillator materials for each layer and the key parameters of metal filters. The optimal selection of scintillators for each detector layer was achieved by comparing the energy deposition response characteristics of different candidate scintillators. For the problem of insufficient distinction in energy response between adjacent layers, the necessity of introducing metal filters between layers to regulate the incident photon spectrum was verified, and the material and thickness parameters of the filters were determined through simulations. Ultimately, the simulation verification of the overall energy deposition distribution of the detector confirmed that each detection layer exhibits unique and significantly distinguishable energy response characteristics in different incident X-ray energy intervals [22]. This five-layer structure can realize X-ray imaging at five different energies, thereby completing the core design verification for the multi-energy detection performance of the detector [69].

For the pyrolysis combined with physical separation process of LiFePO_4_ batteries, the XRD technology was applied to identify the core phase composition of the recycled raw materials and verify whether the pyrolysis process would damage the crystal structure of LiFePO_4_ [47]. The X-ray diffraction (XRD) pattern identified the main crystalline phase of the cathode active materials in spent LiFePO_4_ batteries as LiFePO_4_, laying a phase foundation for the subsequent research on the battery recovery process. The thermogravimetry-differential scanning calorimetry (TG-DSC) curves further revealed that the pyrolysis behavior of spent LiFePO_4_ batteries could be divided into four stages, and defined 500–654.6 °C as the suitable pyrolysis temperature range. Within this range, the effective decomposition of organic components, including electrolyte and binder, could be realized, while the thermal decomposition of LiFePO_4_ was avoided. Fourier transform infrared (FTIR) spectra at different pyrolysis temperatures showed that the characteristic peak of hydrogen bonding, which provides the adhesive force between active materials and current collectors, gradually weakened with the rise of pyrolysis temperature and completely disappeared at 550 °C, confirming that this temperature allows the complete dissociation of active materials from current collectors. Furthermore, the XRD pattern of the cathode active materials after pyrolysis at 550 °C for 2 h verified that this pyrolysis condition would not alter the phase composition of LiFePO_4_ and its crystal structure remained intact, thereby providing a structural guarantee for the regenerative utilization of the cathode active materials.

**Figure 8:**
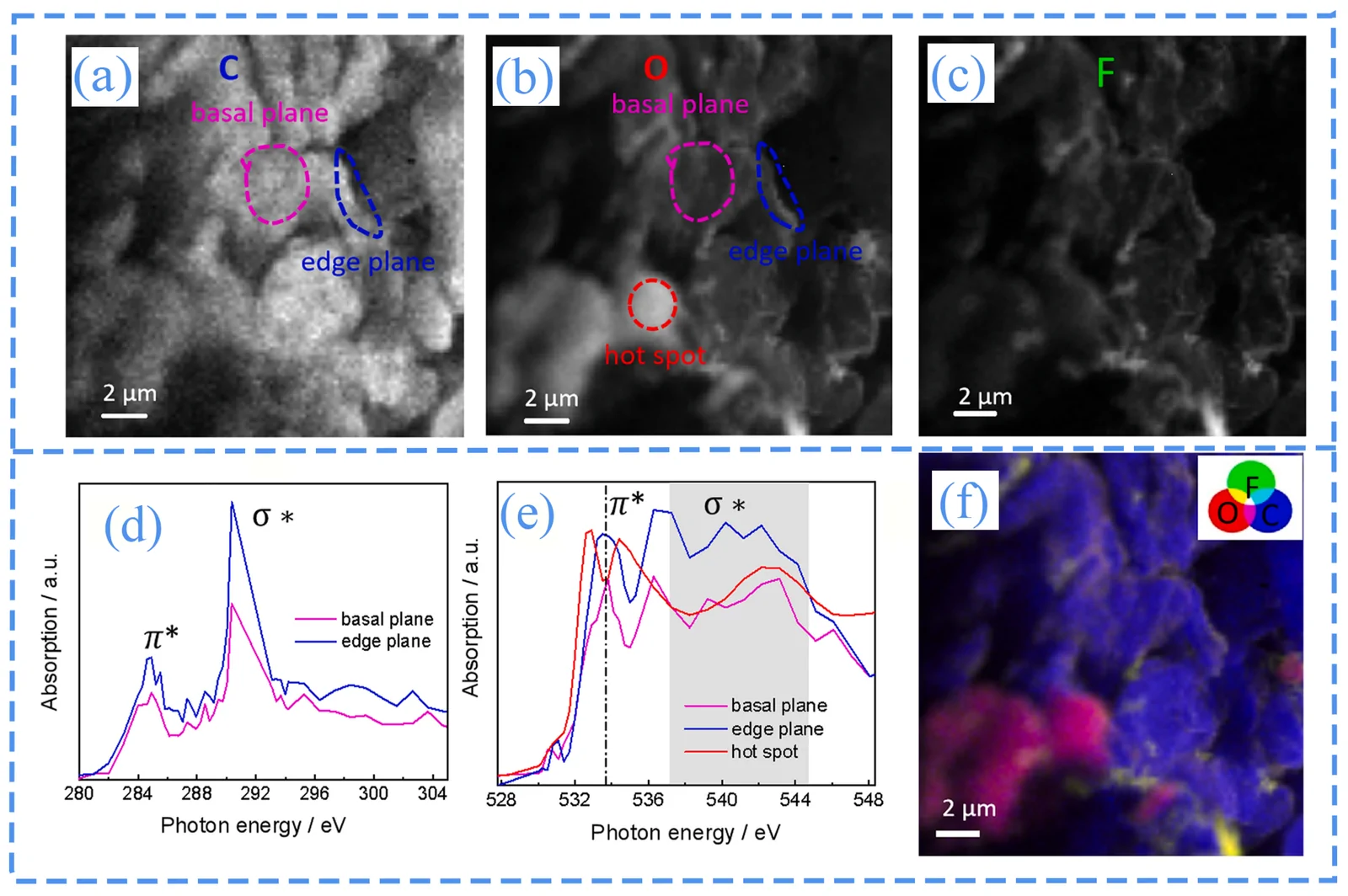
High resolution XRF elemental mapping for C (**a**), O (**b**) and F (**c**) from a degraded graphite anode under 710 eV photon excitation, and rescaled RGB (O, F, C) code composite mapping (**f**); (**d**) C K-edge XRF-STXM XANES spectra selected from (**a**) at basal and edge plane; (**e**) O K-edge XANES selected from (**d**) at hot spot, basal and edge plane [72].

In Figure 8 Zhu achieved high-resolution spatial chemical imaging via X-ray fluorescence scanning transmission X-ray microscopy [72], and revealed the spatial heterogeneity of the solid electrolyte interphase (SEI) by combining it with X-ray absorption near-edge structure (XANES).

Particle characteristics of electrode sheets from spent lithium-ion batteries are detected via sensors, combined with computer algorithm-based decision-making, and then sorted by mechanical devices such as high-speed air valves. Its core value lies in the early removal of invalid components, including copper–aluminum foil impurities and separator debris, thereby reducing energy consumption in subsequent processes like dismantling and leaching, reagent consumption, and wastewater treatment costs, as well as lowering the overall cost of the recycling process. Industrially, it is mainly divided into belt sorters and chute sorters, with the process consisting of five steps: electrode sheet particle pretreatment involving screening and surface degumming → material conveying → sensor detection → data processing → mechanical separation.

DE-XRT is derived from airport luggage security inspection technology. In the field of sorting electrode sheets from spent lithium-ion batteries, it achieves component differentiation through the interaction between dual-energy X-rays and substances. X-ray sources such as 160 kV tungsten target sources emit rays of specific wavelengths. When the rays penetrate the electrode sheet particles in the detection zone, substances with high atomic numbers exhibit stronger X-ray absorption, while substances with low atomic numbers show weaker absorption. Dual-line scanning scintillation detectors on both sides of the conveyor belt receive high- and low-energy X-ray signals separately and convert them into grayscale images, where dark areas indicate high absorption and light areas represent low absorption [69]. The thickness difference of electrode sheet particles is corrected using aluminum filters, and “pseudocolor images” are generated by combining pre-calibration algorithms. Particles are distinguished solely based on atomic weight differences, enabling the detection of internal inclusions in electrode sheet particles. This overcomes the limitation of optical sensors that can only detect surface characteristics of particles.

**Figure 9:**
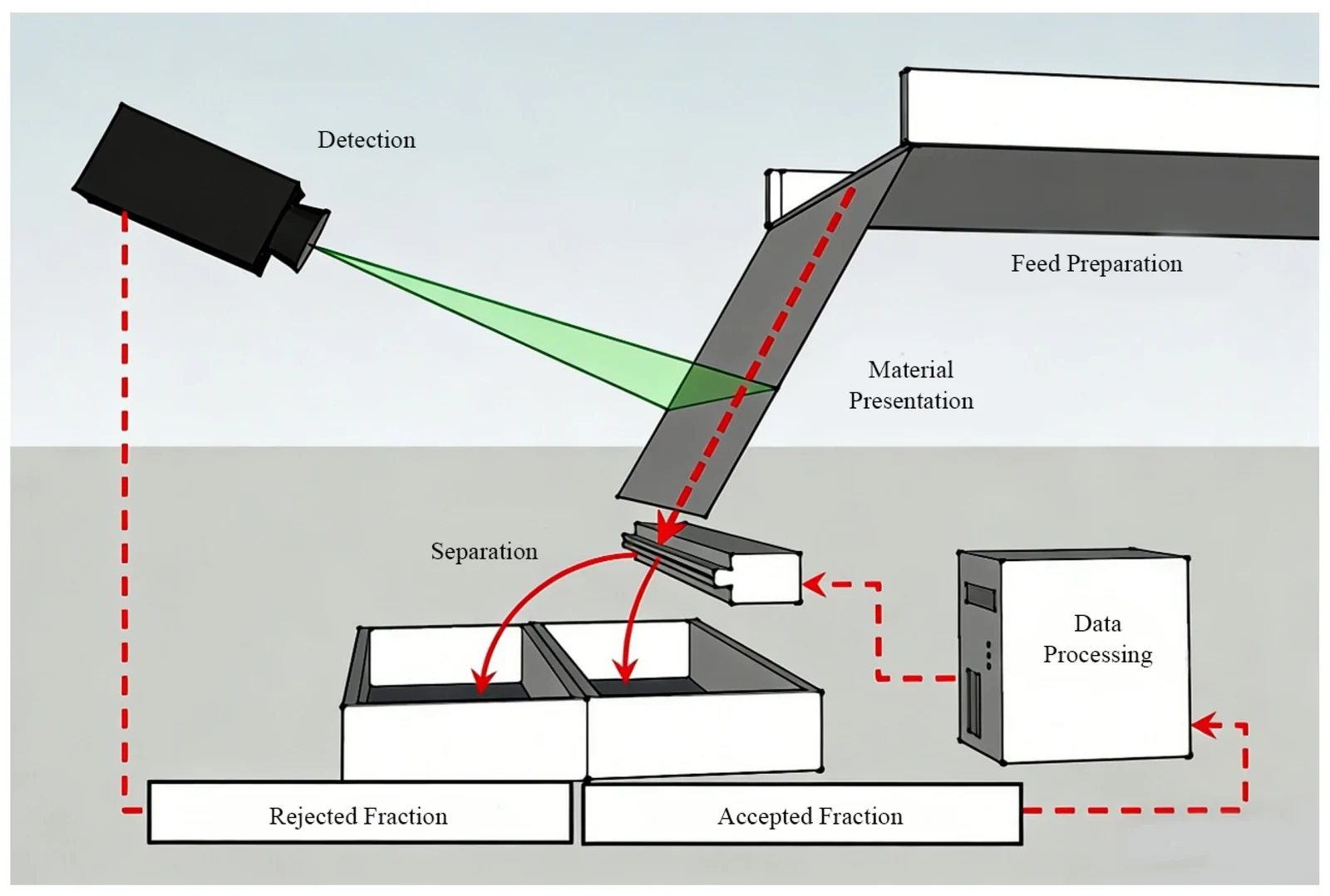
Schematic illustration of a chute-type sorter [75].

In the operation process of the chute-type sensor sorter, the materials first enter the Feed Preparation stage. During this phase, pretreatment operations such as screening and surface cleaning are carried out on the materials. The purpose is to remove impurities and excessively large or small particles from the feedstock, ensuring that the material properties are suitable for subsequent detection procedures and thus greatly improving the accuracy of detection results. After completing pretreatment, the materials immediately enter the Material Presentation stage [73]. Guided by the chute, the materials are uniformly and stably conveyed to the detection zone, which ensures the continuity and consistency of the materials during the detection process. When the materials reach the detection zone, the Detection stage is officially initiated. Taking a sorter adopting DE-XRT technology as an example, the X-ray detector in its detection device conducts a full-range scan of the passing materials. By leveraging the differences in X-ray absorption levels among various substances, the detector acquires information regarding the composition and characteristics of the materials. The signals collected by the detector, such as X-ray attenuation data, are transmitted to the Data Processing module. In this module, the system employs specific algorithms to conduct in-depth analysis of the signals, and compares the analysis results with preset target parameters to accurately determine whether the materials meet the required standards [74]. As shown in Figure 9, based on the judgment results obtained from data processing, the Separation stage is activated. If a material is identified as non-compliant, i.e., classified as the Rejected Fraction, the system will control mechanical devices such as high-speed air valves to alter the movement trajectory of the material, diverting it to a designated collection area. In contrast, the materials judged as qualified, namely the Accepted Fraction, will continue moving along their original path. The entire workflow of the chute-type sensor sorter fully embodies the core logic of sensor-based sorting technology: rapid identification and intelligent separation. It enables accurate screening of large quantities of materials in a short period, removes unqualified materials in advance, significantly reduces the processing load of subsequent procedures such as grinding and flotation, effectively lowers production costs, and improves production efficiency and resource utilization rates [66].

### 4.2. Color Sorting

Analogous to XRT, color/optical sorting crosses from conventional to intelligent when camera signals are analyzed by deep learning models rather than pre-programmed color threshold rules. The distinction lies in the decision algorithm: rule-based RGB thresholding is conventional physical separation; learned feature extraction from color/texture images is intelligent sorting.

**Figure 10:**
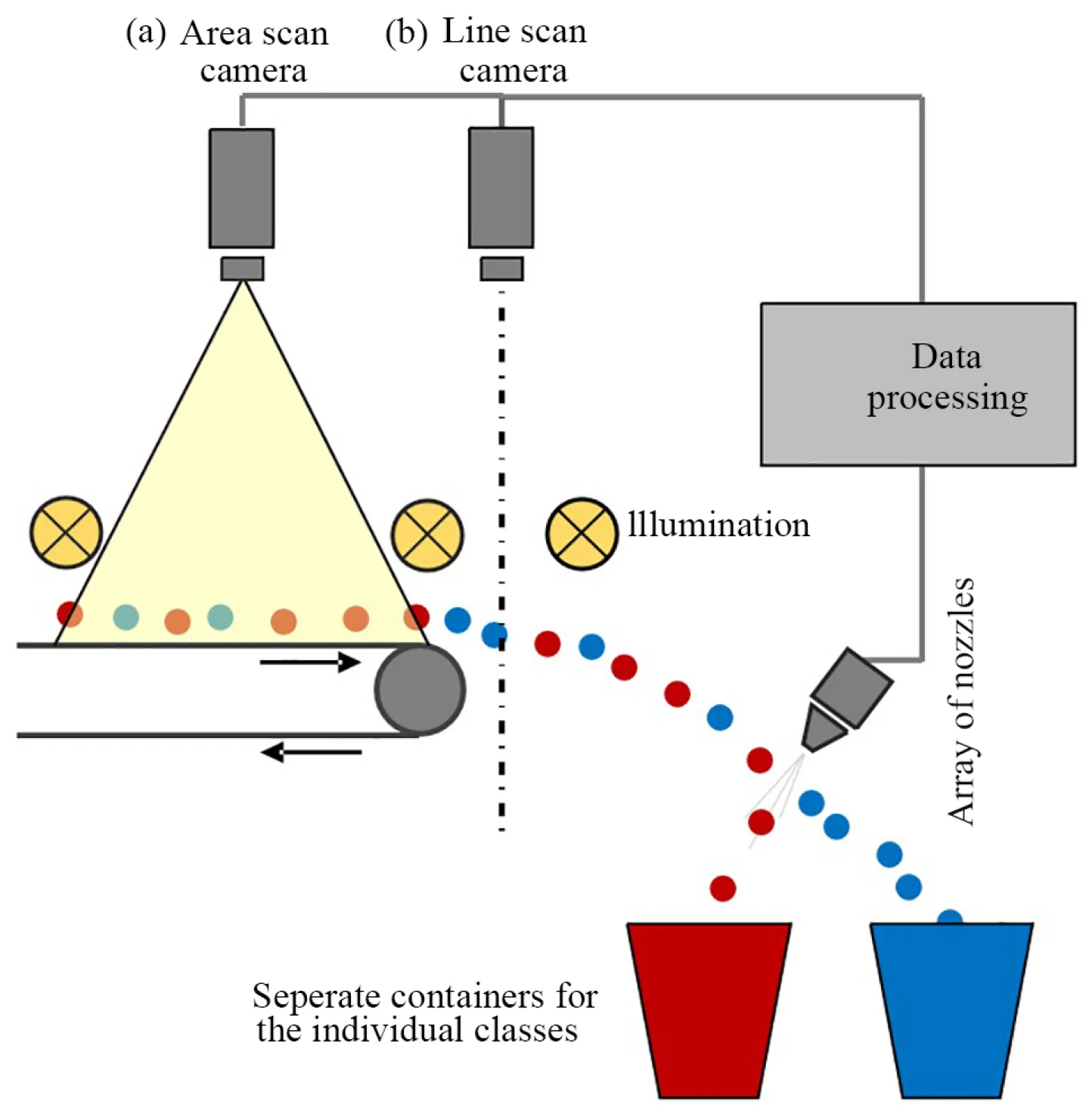
Illustration of an optical belt sorter, which can be equipped with a conventional line scan camera (**b**) or an area scan camera (**a**) [67].

A color sorter is an automated production device for item sorting based on color sorting technology. According to the number of sorting passes items undergo during classification, it is categorized into single-pass color sorters and multi-pass ones with a recheck function. A single-pass color sorter completes classification in one pass, whereas a multi-pass sorter requires at least two passes for certain materials [66]. Generally, multi-pass color sorters exhibit higher sorting precision than single-pass counterparts, with slight structural differences but an identical sorting principle. Figure 10 presents the structure and components of a single-pass color sorter, which mainly comprises an automatic feeding system, a color sorting system, a background adjustment system, a light source control system, a dust cleaning system, and a main control system [76].

Beyond hardware configuration, the operational dynamics of optical belt sorters have been systematically modelled by Pieper et al. [67] using a coupled discrete element method and computational fluid dynamics (DEM-CFD) approach. Their numerical model was extensively validated against experiments and demonstrated the ability to accurately describe sorting performance under various operational parameters, providing a valuable tool for optimizing belt sorter design for battery electrode sorting applications.

### 4.3. Machine Vision and Deep Learning-Based Sorting

#### 4.3.1. Traditional Machine Vision Sorting

Traditional visual inspection for electrode sorting relies on hand-crafted features. It suffers from poor adaptability to lighting changes, weak discrimination between similarly colored materials, difficult trade-offs between over-detection and under-detection, and low scalability for new defect types.

Artificial intelligence (AI), especially deep learning, has emerged as a promising solution. AI can automate battery identification, material characterisation, and robotic disassembly, improving efficiency and reducing human error. AI/ML techniques significantly improve automated sorting and disassembly, enable accurate state-of-health assessment, and reduce chemical consumption in recycling processes [77].

#### 4.3.2. Deep Learning-Enabled Intelligent Sorting

**Figure 11:**
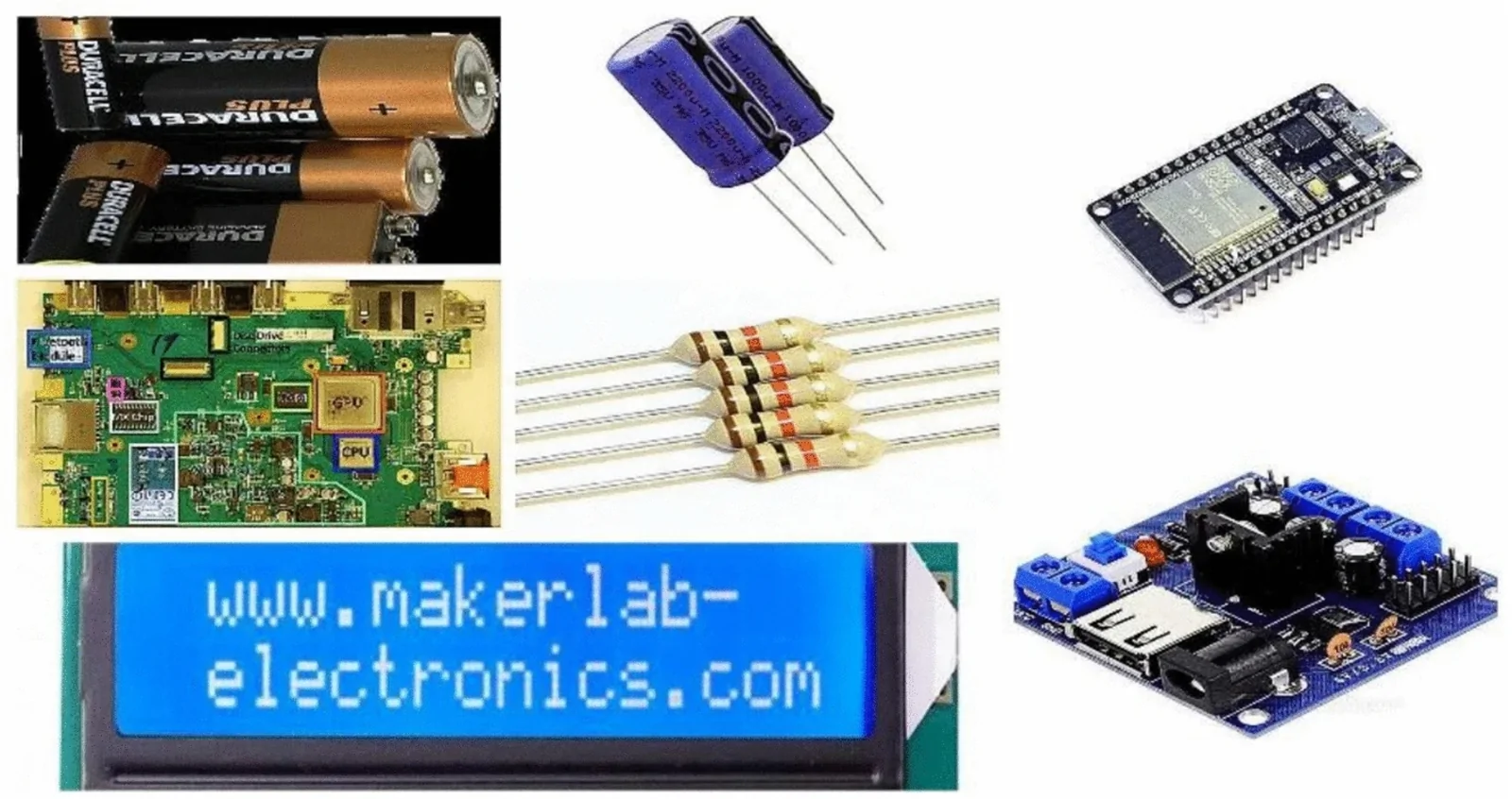
Advancing e-waste classification with customizable YOLO based deep learning models [78].

Deep learning models, particularly YOLO and convolutional neural networks (CNNs), have been widely applied in general waste sorting. For example, YOLOv5 has been used to classify plastics, paper, glass, and metals. It is shown in Figure 11 that in e-waste sorting, Rajeev et al. [78] systematically compared YOLOv5, YOLOv7, and YOLOv8 on a dataset of seven e-waste categories. They found that YOLOv8, with its enhanced feature pyramid network and improved CSPDarknet53 backbone, outperformed the other versions in both accuracy and training speed.

These studies provide a solid technical foundation for applying deep learning to battery electrode sorting, where the visual similarity between cathode and anode materials presents an even greater challenge.

#### 4.3.3. Deep Learning for Battery Sorting and Disassembly

For clarity, the deep-learning studies relevant to electrode recycling are grouped into three distinct application scenarios: (i) whole-battery detection in mixed e-waste, which occurs upstream of dismantling; (ii) manufacturing-defect inspection of pristine electrode sheets; and (iii) direct sorting of crushed electrode particles, which is the central focus of this review. The first two scenarios are discussed here primarily for their architectural transfer value, since direct studies of scenario (iii) remain scarce, as summarized in Table 4.

Recent studies have specifically addressed battery sorting. Lai et al. [23] reviewed machine vision for appearance-based sorting of retired batteries. Antony Jose et al. [79] highlighted the integration of computer vision, spectroscopy, and X-ray imaging with deep learning for precise material characterisation, while noting challenges in data quality and scalability [80].

Focusing on X-ray-based detection, Antony et al. [79] evaluated deep learning for battery recognition in X-ray images of waste electrical and electronic equipment (WEEE). Using 532 devices, they achieved a true positive rate of 91% for battery-containing devices and an average precision of 85% for distinguishing six battery types [79]. This demonstrates the feasibility of combining XRT with deep learning for battery identification.

Koyanaka and Kobayashi further developed an in-line sorting system with a three-stage deep learning architecture for battery detection in e-waste. The system first estimates the e-waste type, then applies a pre-trained network to detect hidden batteries, and finally uses a post-network to recover missed detections. It achieved 96.7% accuracy for trained categories and 77.0% for unseen categories, outperforming single-network baselines [70]. This multi-stage approach is highly relevant for electrode sorting where overlapping fragments and variable surface conditions are common.

**Figure 12:**
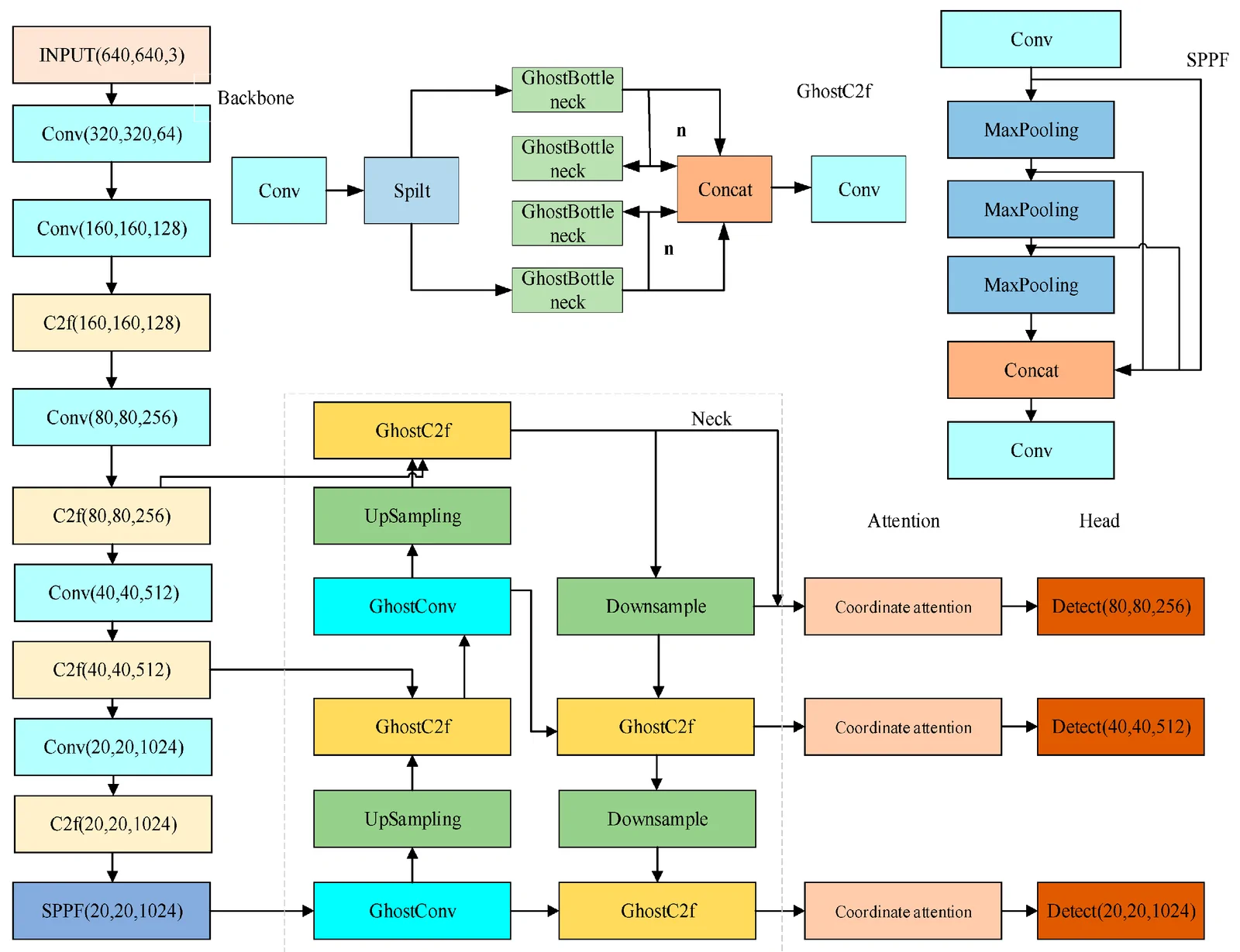
YOLOv8-GCE structure [81].

Figure 12 depicts the YOLOv8 in the specific domain of electrode defect detection, Zhou et al. [81] proposed a YOLOv8-based method for real-time lithium-ion battery electrode defect detection. By introducing GhostConv, a coordinate attention mechanism, and EIoU loss, their improved model increased precision by 2.4%, recall by 2.3%, and mAP by 1.4% while maintaining high inference speed. This work confirms that modified YOLOv8 can meet industrial demands for electrode quality inspection, and the same principles can be adapted for sorting applications.

It is important to emphasize that the studies summarized above address tasks adjacent to—but distinct from—the direct sorting of crushed electrode particles. Battery-level detection, bulk e-waste classification, and electrode manufacturing-defect inspection operate on whole devices, uniform pristine sheets, or intact components, whose visual signatures differ substantially from the irregular, binder-contaminated, partially delaminated particles produced by electrode crushing. Consequently, the transfer of these architectures to electrode material sorting is plausible but remains to be experimentally validated; statements on the applicability of AI-based sorting to crushed electrodes should therefore be read as extrapolations rather than established findings, as formalized in Table 4.

**Table 4:** Applicability of deep-learning vision studies to electrode material sorting: task-level categorization.

Task Category	Definition	Image Features	Typical Datasets	Key Metrics	Applicability to Electrode Sorting	Representative Studies
Battery-Level Detection	Identify whole batteries in mixed e-waste streams prior to dismantling	X-ray transmission images; whole-device shapes; metallic enclosures	532-1000+ WEEE X-ray images; custom industrial datasets	TPR; average precision; per-type accuracy	INDIRECT: Upstream stage; CNN architectures transferable but visual features differ substantially from crushed electrode particles	Sterkens et al. [26]; Koyanaka & Kobayashi [70]
E-Waste Sorting	Classify bulk e-waste items into material categories (plastics, metals, PCBs, batteries)	RGB images of whole items; color, texture, shape; conveyor-belt illumination	Public e-waste datasets (7 categories); custom industrial datasets	Classification accuracy; mAP@0.5; inference FPS	INDIRECT: Demonstrates YOLO architectures transferable to electrode sorting; visual domain differs substantially	Rajeev et al. [70]
Electrode Defect Inspection	Detect manufacturing defects (pinholes, cracks, thickness variation) on pristine electrode sheets	High-resolution optical/SEM images; uniform background; regular coating texture	Proprietary manufacturing QC datasets; line-scan camera images	Precision; recall; mAP; defect-level IoU; inference latency	PARTIALLY: YOLOv8 attention mechanisms (GhostConv, CA, EIoU) are transferable; visual features differ from crushed fragments	Zhou et al. [81]
Electrode Material Sorting	Classify liberated electrode particles (cathode vs. anode; LFP vs. NMC vs. LCO vs. LMO) after crushing and binder removal	RGB/XRT/NIR images of irregular particulate mixtures; variable particle size, shape, surface contamination, delamination degree	NO standardized public dataset exists (critical gap identified in Section 4.5)	Sorting purity (%); recovery (%); Throughput (kg/h); false rejection rate; energy (kWh/t)	DIRECTLY RELEVANT: Core application domain of this review; all future DL-based sorting systems should be validated on this task	Park et al. [82]; Anticoi et al. [83]; Mistry et al. [84]

### 4.4. Technology Comparison

Single sensors have inherent limitations. Multi-sensor fusion integrates complementary information from different sensors to achieve higher accuracy and robustness.

#### 4.4.1. Sensor Types and Fusion Strategies

Common sensors for electrode sorting include:

RGB vision—fast, low-cost, captures colour, texture, shape; sensitive to lighting and surface contamination. XRT—detects atomic number (Z) and internal features; unaffected by surface dust; high capital cost. Hyperspectral/NIR –identifies chemical composition, but expensive and data-intensive. LIBS—elemental analysis; destructive and slower [53].

Fusion can be performed at three levels: data-level (pixel-wise alignment), feature-level, or decision-level. Integrating machine learning and computer vision into a multi-sensor framework can overcome the drawbacks of single sensors and improve sorting efficiency [85].

#### 4.4.2. XRT + RGB Synergy

XRT and RGB are highly complementary: XRT provides material identification based on atomic number, while RGB gives surface color and morphology. Their combination achieves “internal + external” characterisation. In mineral processing, dual-energy XRT (DE-XRT) combined with visible vision has shown clear advantages for intelligent pre-concentration. This approach can be directly adapted to battery electrode sorting [86].

The studies by Sterkens et al. [26] and Koyanaka & Kobayashi [70] provide direct evidence that XRT combined with deep learning can effectively detect and classify batteries in e-waste, which is a strong indicator that XRT + RGB fusion would work for electrode sorting.

#### 4.4.3. Hyperspectral and NIR Imaging

**Figure 13:**
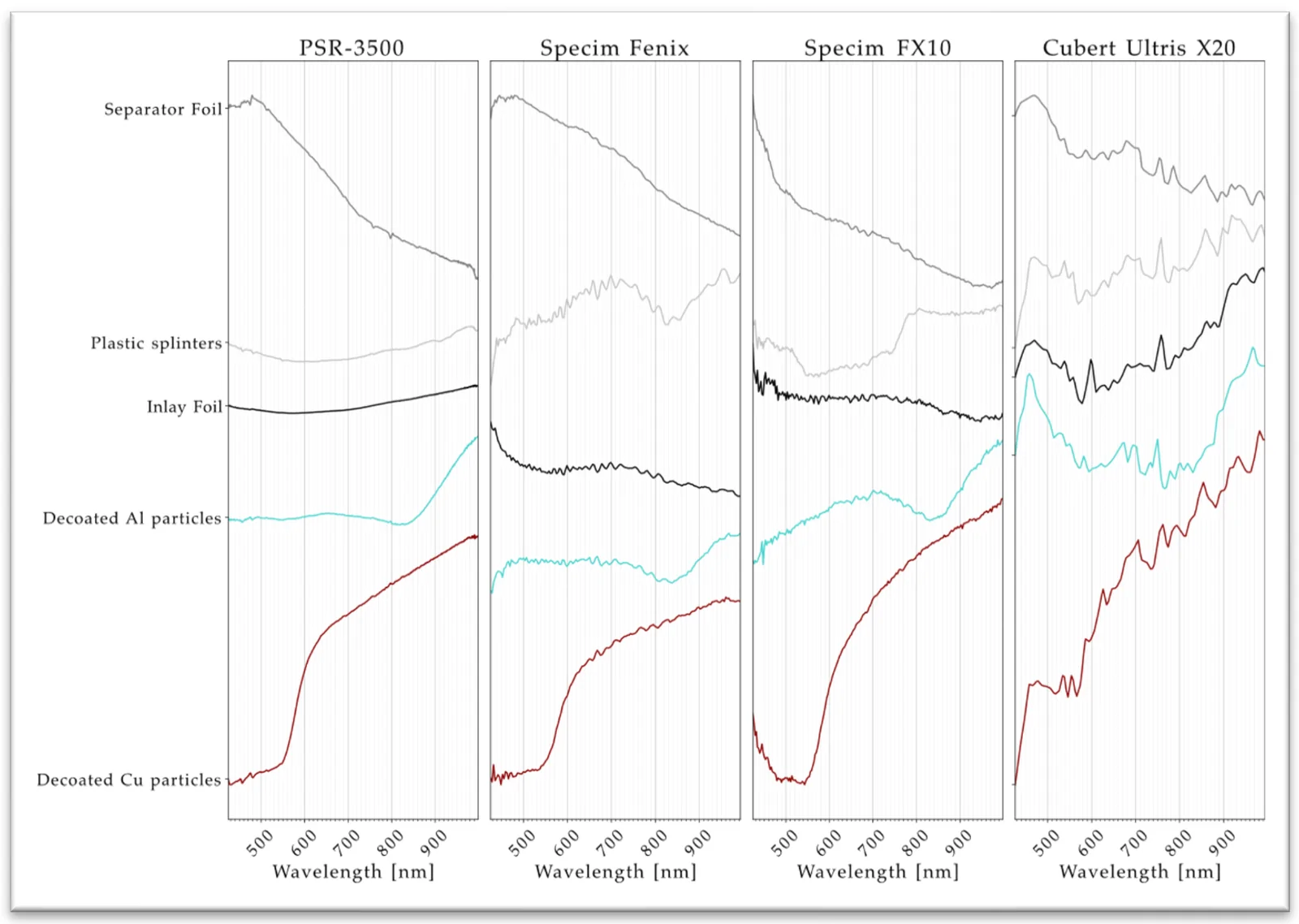
Representative reflectance spectra of decoated Al and Cu particles as well as of plastic components (i.e., separator foil, plastic splinters, and inlay foil) obtained from the Spectral Evolution PSR-3500 are compared to averaged HSI results recorded by the Specim Aisa Fenix, Specim FX10, and Cubert Ultris X20 in the range of 400–1000 nm. The discriminative features, a step at around 580 nm for Cu and a minimum at around 830 nm followed by a steeper slope for Al, are reproduced by all HSI measurements [27].

Hyperspectral imaging acquires a “spectral cube”, enabling chemical identification of cathode materials. Richter et al. provided systematic spectral characterisation of battery components from different recycling streams, creating a valuable database for combining spectroscopy with deep learning, the data are presented in Figure 13 [27]. However, high cost and slow processing still limit industrial application. Similarly, Qiu et al. [87] demonstrated the feasibility of combining deep learning with photofluorescence for uranium ore sorting. By exploiting the inherent fluorescence of uranium ore under ultraviolet light coupled with two distinct deep learning models, the proposed method achieved effective separation of ore from waste. However, the study also noted that image quality in fluorescence-based sorting is easily affected by environmental factors such as dust and noise, leading to degradation and reduced accuracy—a challenge that is equally relevant for hyperspectral imaging in battery electrode sorting.

### 4.5. Chapter Summary

This chapter reviews intelligent sorting technologies and presents a structured gap analysis. XRT sorting, color sorting, deep-learning-based visual recognition, and multi-sensor fusion are systematically examined. In XRT sorting, multi-energy detector design and integration with pyrolysis pretreatment are described. In deep learning, the limitations of traditional machine vision are first analyzed, followed by successful applications of YOLO models in solid waste and e-waste sorting, with recent studies on battery-specific sorting summarized. A structured gap analysis identifies five critical unresolved challenges: (1) absence of standardized multi-sensor electrode datasets; (2) lack of real-time inference benchmarks at industrial throughput; (3) limited cross-chemistry model generalizability; (4) insufficient investigation of multimodal sensor fusion strategies; and (5) absence of community-standard evaluation metrics. Multi-sensor fusion is identified as a key direction to overcome single-sensor limitations. The chapter concludes that while deep learning shows substantial promise, bridging the identified gaps requires coordinated efforts in dataset construction, model optimization, and standardized evaluation protocol development.

To translate the general deep-learning advances discussed above into actionable solutions for electrode material sorting, Table 5 decomposes this overarching task into four hierarchical sub-tasks of increasing complexity. For each sub-task, the table specifies the required image features, dataset scale, annotation protocols, and key evaluation metrics, providing a structured blueprint for both dataset construction and model development.

## 5. Conclusions and Future Perspectives

Against the backdrop of global energy conservation, emission reduction, and the rapid growth of the lithium-ion battery industry, the recycling of spent lithium-ion battery electrodes has become a critical link in resource recovery, environmental protection, and the sustainable development of the new energy sector. Three synthesized conclusions emerge from this review. First, no single physical separation technology is sufficient on its own: eddy current and pneumatic separation provide high-throughput bulk pre-concentration but degrade for fine and irregular fragments, electrostatic separation handles fine particles but is humidity-sensitive, and XRT offers the richest discrimination at the highest cost, so industrial recovery necessarily relies on multi-stage circuits in which each unit operation plays a complementary role. Second, intelligent sensor-based sorting is complementary to—rather than a replacement for—conventional separation: conventional modes are industrially mature (TRL 7–9) but operate on fixed thresholds, whereas deep-learning modes (TRL 3–4) offer adaptivity to variable feedstock but remain unvalidated at industrial throughput. Third, the bottleneck for electrode recycling is shifting from material recognition to liberation and data: for the anode, the limiting step is liberation quality rather than detection, and for intelligent sorting, the limiting step is the absence of standardized multi-sensor datasets rather than model architecture itself.

Future research should focus on five core directions, prioritized by their potential impact on industrial deployment and the maturity of enabling technologies:

These five directions are not a list of general aspirations. They follow from three cross-cutting constraints that recur throughout this review. First, a data bottleneck: the accuracy of deep-learning sorting is ultimately limited by the labelled, multi-sensor, multi-chemistry datasets on which it is trained, and no community-standard dataset yet exists for crushed electrode materials. Second, a throughput–accuracy tension: laboratory demonstrations report high accuracy, but few are validated at the multi-tonne-per-hour throughput and under the dust, vibration, and feed variability of industrial sorting lines, where the economic case for sensor-based sorting must ultimately be made. Third, a missing design–recycling feedback loop: physical separation is bounded by upstream design choices (binder chemistry, current-collector thickness, cell architecture) that are made without regard to end-of-life liberation, so recyclers currently optimize around designs they cannot influence. Each direction below identifies the blocking obstacle and the concrete next step required to remove it.

1. Lightweight deep learning models for edge deployment. Current YOLOv8-based sorting systems rely on GPU-equipped workstations that increase capital and energy costs. Future work should investigate model compression techniques to deploy YOLO-based classifiers on edge-AI devices while maintaining >90% classification accuracy at conveyor-belt throughput (>1 t/h). Preliminary studies in general waste sorting suggest that INT8-quantized YOLOv8-nano models can achieve real-time inference (<10 ms/frame) with <3% accuracy degradation compared to FP32 equivalents, but validation on electrode-specific datasets is lacking.
2. Transfer learning across battery chemistries and form factors. Deep learning models trained on one battery type exhibit significant performance degradation when applied to other chemistries or physical formats. Domain adaptation and few-shot learning techniques should be systematically evaluated to quantify cross-chemistry generalization and to establish the minimum number of training samples required for effective model adaptation to a new battery stream.
3. Systematic multimodal sensor fusion. While individual sensor modalities have been independently evaluated for electrode sorting, systematic studies comparing data-level, feature-level, and decision-level fusion strategies on identical feedstock are absent. Future work should establish standardized multi-sensor datasets and benchmark fusion architectures, with particular attention to the XRT + RGB combination, which offers complementary internal and external characterization at acceptable cost.
4. Design-for-recycling and cross-sector collaboration. Physical separation efficiency is ultimately bounded by upstream design choices—the binder, current-collector thickness, and cell architecture—that are rarely optimized for end-of-life liberation. Co-optimization between battery manufacturers and recyclers is required to close the design–recycling loop, ensuring that design-for-recycling modifications do not compromise electrochemical performance. Concretely, this entails quantifying the separation-cost penalty of current designs and establishing design guidelines, such as binder selection, that measurably improve liberation without sacrificing cycle life.
5. Standardized evaluation protocols and benchmark datasets. The absence of community-standard metrics, benchmark datasets, and evaluation protocols for electrode sorting prevents reproducible comparison across studies. Future work should establish: (a) open-access, multi-chemistry, multi-sensor electrode image datasets with expert-verified ground-truth labels; (b) standardized metrics beyond classification accuracy, including sorting purity, recovery, throughput (kg/h), and energy consumption (kWh/t); and (c) common evaluation protocols specifying train/test split criteria, cross-validation procedures, and statistical significance reporting.

## Figures and Tables

**Table 1:** Components of a lithium-ion battery with LCO, NMC, and LFP cathodes [29].

Component	LCO (%)	NMC (%)	LFP (%)
cathode active material	41	26	25
anode active material	18	15	13
housing	17	22	27
electrolyte	10	10	16
anode Cu foil	7	17	10
cathode Al foil	4	7	6
separator	3	3	3

**Table 2:** Comparative evaluation of physical separation technologies for spent LIB electrodes.

Technology	Target	Principle	Efficiency (%)	Advantages	Limitations	Energy/Chemicals	TRL
Eddy current	Al, Cu foils	Induced eddy current repulsion	85–95 [53]	High throughput, dry	size > 2 mm, shape-sensitive	Low (electric)	9
Electrostatic	Conductive vs. non-conductive	Corona charging + drum	80–90 [56]	Works for fine particles (0.1–5 mm)	Humidity sensitive, pre-drying needed	Very low (HV)	8
Pneumatic	Density/shape	Airflow + gravity	93–96 [63]	Simple, low cost, high capacity	Poor for similar-density, dust	Moderate (air)	9
Color sorter	Color-different materials	RGB camera + air jet	85–95 [66,67]	Fast, non-contact	Sensitive to lighting, surface contamination	Low (LED + air)	8–9
XRT sorting	Atomic number (Z)	Dual/multi-energy X-ray transmission	90–98 [22,67]	Detects internal features, unaffected by dust	High capital cost, radiation safety	Moderate (X-ray)	7–8

**Table 5:** Taxonomy of visual detection and sorting tasks relevant to LIB electrode recycling, distinguishing four task categories by image features, datasets, metrics, and degree of direct applicability to electrode material sorting.

Task Category	Image Features	Dataset Requirements	Key Evaluation Metrics	Direct Applicability to Electrode Sorting
Binary Classification (Sorting vs. Reject)	Global color, texture, and shape descriptors; RGB histograms; simple morphological features	~10,000–50,000 labeled images; binary labels (accept/reject); balanced or imbalanced as per process requirements	Accuracy, precision, recall, F1-score, AUC-ROC, sorting purity (%), recovery (%)	High—directly maps to industrial sorting decisions; can be deployed with simple CNNs or ML classifiers; minimal annotation cost
Multi-Class Chemistry Identification	Fine-grained color variations, surface oxidation patterns, minor texture differences; XRT attenuation coefficients; NIR spectral signatures	≥3500 samples per chemistry as per Gap 1 specs (7 chemistries × 500 samples × 3 SOH); pixel-level multi-sensor registration; inter-annotator κ ≥ 0.8	Per-class precision/recall, macro/micro F1-score, confusion matrix, sorting purity per chemistry, false rejection rate per chemistry	Direct—enables chemistry-specific downstream processing (e.g., hydrometallurgical separation); requires largest dataset and most rigorous labeling
Degradation and Anomaly Detection	Binder residue maps (SEM/EDS co-registered), crack patterns, surface contamination, delamination degree indicators	Subset of multi-class dataset with SEM/EDS validation on 5% subsample; labels for delamination degree (fully/partially/poorly liberated), SOH states	Delamination classification accuracy, anomaly detection F1, correlation with SOH, false alarm rate, inference time per particle	Medium-High—critical for quality control and process optimization; can inform adaptive sorting parameters based on material condition
Particle-Level Instance Segmentation	Per-particle boundaries, size distribution, shape descriptors (aspect ratio, circularity), occlusion handling; fiducial markers for multi-sensor alignment	Pixel-wise mask annotations; four size fractions (0.5–2, 2–5, 5–15, >15 mm); size distribution histograms per chemistry; train/val/test: 70/15/15 stratified	mAP@0.5, mAP@0.5:0.95, per-size-fraction F1-score, throughput (kg/h per m), model size (MB) and FLOPS, inference time	Medium—provides detailed particle-level information for high-precision sorting; computationally intensive; most applicable to conveyor-belt XRT systems

## Data Availability

The original contributions presented in this study are included in the article. Further inquiries can be directed to the corresponding authors. No new data were created or analyzed in this study.

## References

[1] Gu K., Chang J., Mao X., Zeng H., Qin W., Han J. (2022). Efficient Separation of Cathode Materials and Al Foils from Spent Lithium Batteries with Glycerol Heating: A Green and Unconventional Way. J. Clean. Prod..

[2] Wang J., Wang H., Zhang G., Ma X., Liu Y., Hao J., Xie W., He Y. (2025). A Review of Research Progress on Combined Pyro-Hydrometallurgical Technology for Spent Lithium-Ion Batteries. Chem. Eng. J..

[3] Sommerville R., Zhu P., Rajaeifar M.A., Heidrich O., Goodship V., Kendrick E. (2021). A Qualitative Assessment of Lithium Ion Battery Recycling Processes. Resour. Conserv. Recycl..

[4] Cui K., Zhao M.-C., Li Y., Atrens A., Zhang F. (2025). Recycling of Spent Lithium Iron Phosphate Batteries: Research Progress Based on Environmental Protection and Sustainable Development Technology. Sep. Purif. Technol..

[5] Luo B., Xu B., Yan Q., Zhou Y., Dong Z., Huang X., Zhao Z. (2025). Advances and Challenges in Recycling Spent LiFePO_4_ Batteries. Sep. Purif. Technol..

[6] Zhao T., Choi Y., Wu C., Zhang Z., Wang C., Liu D., Xu W., Huang H., Huo X., Zhao W. (2024). A Review on Direct Regeneration of Spent Lithium Iron Phosphate: From Waste to Wealth. Sci. Total Environ..

[7] Harper G., Sommerville R., Kendrick E., Driscoll L., Slater P., Stolkin R., Walton A., Christensen P., Heidrich O., Lambert S. (2019). Recycling Lithium-Ion Batteries from Electric Vehicles. Nature.

[8] Zhong X., Han J., Chen L., Liu W., Jiao F., Zhu H., Qin W. (2021). Binding Mechanisms of PVDF in Lithium Ion Batteries. Appl. Surf. Sci..

[9] Zhu H., Bai Y., Zu L., Bi H., Wen J. (2023). Separation of Metal and Cathode Materials from Waste Lithium Iron Phosphate Battery by Electrostatic Process. Separations.

[10] Zhao T., Mahandra H., Marthi R., Ji X., Zhao W., Chae S., Traversy M., Li W., Yu F., Li L. (2024). An Overview on the Life Cycle of Lithium Iron Phosphate: Synthesis, Modification, Application, and Recycling. Chem. Eng. J..

[11] Randau S., Weber D.A., Kötz O., Koerver R., Braun P., Weber A., Ivers-Tiffée E., Adermann T., Kulisch J., Zeier W.G. (2020). Benchmarking the Performance of All-Solid-State Lithium Batteries. Nat. Energy.

[12] Reddy M.V., Mauger A., Julien C.M., Paolella A., Zaghib K. (2020). Brief History of Early Lithium-Battery Development. Materials.

[13] Mrozik W., Rajaeifar M.A., Heidrich O., Christensen P. (2021). Environmental Impacts, Pollution Sources and Pathways of Spent Lithium-Ion Batteries. Energy Environ. Sci..

[14] Han C., Gao P., Yuan S., Wen G., Ding H., Bai Z., Lei S. (2025). Comprehensive Study of Pyrolysis of Spent Lithium Iron Phosphate Batteries. Sep. Purif. Technol..

[15] Lei S., Sun W., Yang Y. (2024). Comprehensive Technology for Recycling and Regenerating Materials from Spent Lithium Iron Phosphate Battery. Environ. Sci. Technol..

[16] Xu S., Cai J., Liu T., An G., Li Y., Wang Z., Liu J., Li Y., Yan X. (2025). A Review on Electrochemical Recycling of Spent Lithium-Ion Batteries Electrode Materials: Technology Innovation-Driven Resource Closed-Loop Systems and Sustainable Development. Sep. Purif. Technol..

[17] Nong C., Yuan H., Liu Z., Liu H., Han J., Gu J., Chen Y. (2026). Hydrometallurgical Recycling Technologies for Spent LiFePO_4_ Batteries: Recent Advances and Challenges. Renew. Sustain. Energy Rev..

[18] Brückner L., Frank J., Elwert T. (2020). Industrial Recycling of Lithium-Ion Batteries—A Critical Review of Metallurgical Process Routes. Metals.

[19] Wu J., Zheng M., Liu T., Wang Y., Liu Y., Nai J., Zhang L., Zhang S., Tao X. (2023). Direct Recovery: A Sustainable Recycling Technology for Spent Lithium-Ion Battery. Energy Storage Mater..

[20] Bae H., Kim Y. (2021). Technologies of Lithium Recycling from Waste Lithium Ion Batteries: A Review. Mater. Adv..

[21] Kim S., Bang J., Yoo J., Shin Y., Bae J., Jeong J., Kim K., Dong P., Kwon K. (2021). A Comprehensive Review on the Pretreatment Process in Lithium-Ion Battery Recycling. J. Clean. Prod..

[22] Charpentier N.M., Maurice A.A., Xia D., Li W.-J., Chua C.-S., Brambilla A., Gabriel J.-C.P. (2023). Urban Mining of Unexploited Spent Critical Metals from E-Waste Made Possible Using Advanced Sorting. Resour. Conserv. Recycl..

[23] Lai X., Huang Y., Deng C., Gu H., Han X., Zheng Y., Ouyang M. (2021). Sorting, Regrouping, and Echelon Utilization of the Large-Scale Retired Lithium Batteries: A Critical Review. Renew. Sustain. Energy Rev..

[24] Wu Z., Jia W., Wu Y., Hei D., Sun A., Ling Y., Shan Q., Shi C., Zhang J. (2023). Design of a Five-Layers Multi-Energy X-Ray Imaging Detector for Material Sorting. Appl. Radiat. Isot..

[25] Diekmann J., Hanisch C., Froböse L., Schälicke G., Loellhoeffel T., Fölster A.-S., Kwade A. (2017). Ecological Recycling of Lithium-Ion Batteries from Electric Vehicles with Focus on Mechanical Processes. J. Electrochem. Soc..

[26] Sterkens W., Diaz-Romero D., Goedemé T., Dewulf W., Peeters J.R. (2021). Detection and Recognition of Batteries on X-Ray Images of Waste Electrical and Electronic Equipment Using Deep Learning. Resour. Conserv. Recycl..

[27] Richter J., Lorenz S., Kaas A., Fuchs M., Röder C., Peuker U.A., Heitmann J., Gloaguen R. (2024). Spectral Characterization of Battery Components from Li-Ion Battery Recycling Processes. Metals.

[28] Wang H., Qu G., Yang J., Zhou S., Li B., Wei Y. (2022). An Effective and Cleaner Discharge Method of Spent Lithium Batteries. J. Energy Storage.

[29] Golubkov A.W., Fuchs D., Wagner J., Wiltsche H., Stangl C., Fauler G., Voitic G., Thaler A., Hacker V. (2014). Thermal-Runaway Experiments on Consumer Li-Ion Batteries with Metal-Oxide and Olivin-Type Cathodes. RSC Adv..

[30] Windisch-Kern S., Gerold E., Nigl T., Jandric A., Altendorfer M., Rutrecht B., Scherhaufer S., Raupenstrauch H., Pomberger R., Antrekowitsch H. (2022). Recycling Chains for Lithium-Ion Batteries: A Critical Examination of Current Challenges, Opportunities and Process Dependencies. Waste Manag..

[31] Neumann J., Petranikova M., Meeus M., Gamarra J.D., Younesi R., Winter M., Nowak S. (2022). Recycling of Lithium-Ion Batteries—Current State of the Art, Circular Economy, and Next Generation Recycling. Adv. Energy Mater..

[32] Ma X., Ge P., Wang L., Sun W., Bu Y., Sun M., Yang Y. (2023). The Recycling of Spent Lithium-Ion Batteries: Crucial Flotation for the Separation of Cathode and Anode Materials. Molecules.

[33] Werner D., Peuker U.A., Mütze T. (2020). Recycling Chain for Spent Lithium-Ion Batteries. Metals.

[34] Zhao X., Li X., Gao Q., Li X., Wei G., Yan S., Zhu X., Ren Y. (2025). Sustainable and Efficient Recycling Strategies for Spent Lithium Iron Phosphate Batteries: Current Status and Prospect. Sep. Purif. Technol..

[35] Zhong X., Liu W., Han J., Jiao F., Qin W., Liu T. (2020). Pretreatment for the Recovery of Spent Lithium Ion Batteries: Theoretical and Practical Aspects. J. Clean. Prod..

[36] Thompson D.L., Hartley J.M., Lambert S.M., Shiref M., Harper G.D.J., Kendrick E., Anderson P., Ryder K.S., Gaines L., Abbott A.P. (2020). The Importance of Design in Lithium Ion Battery Recycling—A Critical Review. Green Chem..

[37] Zhang G., Du Z., He Y., Wang H., Xie W., Zhang T. (2019). A Sustainable Process for the Recovery of Anode and Cathode Materials Derived from Spent Lithium-Ion Batteries. Sustainability.

[38] Vanderbruggen A., Hayagan N., Bachmann K., Ferreira A., Werner D., Horn D., Peuker U., Serna-Guerrero R., Rudolph M. (2022). Lithium-Ion Battery Recycling─Influence of Recycling Processes on Component Liberation and Flotation Separation Efficiency. ACS EST Eng..

[39] Kong Y., Takaya Y., Córdova-Udaeta M., Tokoro C. (2024). A Comprehensive Approach for the Recycling of Anode Materials from Spent Lithium-Ion Batteries: Separation, Lithium Recovery, and Graphite Reutilization as Environmental Catalyst. Waste Manag..

[40] Vanderbruggen A., Salces A., Ferreira A., Rudolph M., Serna-Guerrero R. (2022). Improving Separation Efficiency in End-of-Life Lithium-Ion Batteries Flotation Using Attrition Pre-Treatment. Minerals.

[41] Sun Q., Zeng K., Chen J., Zhang H., Zeng H. (2026). Flotation Separation in Lithium-Ion Battery Recycling: Challenges and Recent Advances. Adv. Colloid Interface Sci..

[42] Zhang J., Li J., Wang Y., Sun M., Wang L., Tu Y. (2022). Separation of Graphites and Cathode Materials from Spent Lithium-Ion Batteries Using Roasting–Froth Flotation. Sustainability.

[43] Tokoro C., Horiuchi M., Narita A., Kubota A., Takaya Y. (2025). Optimization of Graphite Anode Delamination and Contaminant Separation via Electrical Pulsed Discharge for Direct Recycling. Adv. Powder Technol..

[44] Sargent A.T., Henderson Z., Walton A.S., Spencer B.F., Sweeney L., Flavell W.R., Anderson P.A., Kendrick E., Slater P.R., Allan P.K. (2023). Reclamation and Reuse of Graphite from Electric Vehicle Lithium-Ion Battery Anodes *via* Water Delamination. J. Mater. Chem. A.

[45] Verdugo L., Zhang L., Saito K., Bruckard W., Menacho J., Hoadley A. (2022). Flotation Behavior of the Most Common Electrode Materials in Lithium Ion Batteries. Sep. Purif. Technol..

[46] Li J., Zhang J., Zhao W., Lu D., Ren G., Tu Y. (2022). Application of Roasting Flotation Technology to Enrich Valuable Metals from Spent LiFePO_4_ Batteries. ACS Omega.

[47] Zhong X., Liu W., Han J., Jiao F., Qin W., Liu T., Zhao C. (2019). Pyrolysis and Physical Separation for the Recovery of Spent LiFePO_4_ Batteries. Waste Manag..

[48] Li Y., Deng L., Cao Y., He J., Li G. (2025). A Critical Review on the Direct Regeneration Technologies of Cathode Materials from Spent Lithium Iron Phosphate Batteries. Rare Met..

[49] Chen C., Min Y., Wang Q., Huang Q. (2025). Polyvinylidene Fluoride Binder Removal through Subcritical Methanol for Efficient Liberation of Cathode Materials from Lithium-Ion Batteries. Waste Manag..

[50] Yang Y., Li Y., Guan T., Peng S., Liu N., Cui B., Lu J., Zhang X. (2025). Recovery of Lithium Iron Phosphate Cathode Material from Spent Lithium-Ion Batteries Using Gradient Pyrolysis. Sep. Purif. Technol..

[51] Zhu P., Driscoll E.H., Dong B., Sommerville R., Zorin A., Slater P.R., Kendrick E. (2023). Direct Reuse of Aluminium and Copper Current Collectors from Spent Lithium-Ion Batteries. Green Chem..

[52] Zhang J., Azimi G. (2022). Recycling of Lithium, Cobalt, Nickel, and Manganese from End-of-Life Lithium-Ion Battery of an Electric Vehicle Using Supercritical Carbon Dioxide. Resour. Conserv. Recycl..

[53] Bi H., Zhu H., Zu L., Bai Y., Gao S., Gao Y. (2019). A New Model of Trajectory in Eddy Current Separation for Recovering Spent Lithium Iron Phosphate Batteries. Waste Manag..

[54] Smith Y.R., Nagel J.R., Rajamani R.K. (2019). Eddy Current Separation for Recovery of Non-Ferrous Metallic Particles: A Comprehensive Review. Miner. Eng..

[55] Bi H., Zhu H., Zu L., He S., Gao Y., Gao S. (2019). Pneumatic Separation and Recycling of Anode and Cathode Materials from Spent Lithium Iron Phosphate Batteries. Waste Manag. Res. J. Sustain. Circ. Econ..

[56] Ruan J., Qian Y., Xu Z. (2014). Environment-Friendly Technology for Recovering Nonferrous Metals from e-Waste: Eddy Current Separation. Resour. Conserv. Recycl..

[57] Erdogan C., Contreras C.A., Rastegarpanah A., Chiou M., Stolkin R. (2025). Intent-Driven LLM Ensemble Planning for Flexible Multi-Robot Disassembly: Demonstration on EV Batteries. arXiv.

[58] Bai Y., Zhu H., Zu L., Bi H. (2023). Eddy Current Separation of Broken Lithium Battery Products in Consideration of the Shape Factor. J. Mater. Cycles Waste Manag..

[59] Silveira A.V.M., Santana M.P., Tanabe E.H., Bertuol D.A. (2017). Recovery of Valuable Materials from Spent Lithium Ion Batteries Using Electrostatic Separation. Int. J. Miner. Process..

[60] Lee H., Driscoll E.H., Waters K., Kendrick E., Sommerville R. (2025). Pretreatment and Valorization of Critical Materials from Lithium-Ion Batteries Using Electrostatic and Magnetic Separation. Adv. Energy Sustain. Res..

[61] Hu Z., Liu J., Gan T., Lu D., Wang Y., Zheng X. (2022). High-Intensity Magnetic Separation for Recovery of LiFePO_4_ and Graphite from Spent Lithium-Ion Batteries. Sep. Purif. Technol..

[62] Carlstedt N., Brungi P., Andrei P. (2025). High-Gradient Magnetic Chromatography of Rare-Earth Elements. AIP Adv..

[63] Dong L., Zhang Y., Zhao Y., Peng L., Zhou E., Cai L., Zhang B., Duan C. (2016). Effect of Active Pulsing Air Flow on Gas-Vibro Fluidized Bed for Fine Coal Separation. Adv. Powder Technol..

[64] Granata G., Moscardini E., Pagnanelli F., Trabucco F., Toro L. (2012). Product Recovery from Li-Ion Battery Wastes Coming from an Industrial Pre-Treatment Plant: Lab Scale Tests and Process Simulations. J. Power Sources.

[65] Zhu X., Zhang C., Feng P., Yang X., Yang X. (2021). A Novel Pulsated Pneumatic Separation with Variable-Diameter Structure and Its Application in the Recycling Spent Lithium-Ion Batteries. Waste Manag..

[66] Shatwell D.G., Murray V., Barton A. (2023). Real-Time Ore Sorting Using Color and Texture Analysis. Int. J. Min. Sci. Technol..

[67] Pieper C., Pfaff F., Maier G., Kruggel-Emden H., Wirtz S., Noack B., Gruna R., Scherer V., Hanebeck U.D., Längle T. (2018). Numerical Modelling of an Optical Belt Sorter Using a DEM–CFD Approach Coupled with Particle Tracking and Comparison with Experiments. Powder Technol..

[68] Verdugo L., Zhang L., Etschmann B., Bruckard W., Menacho J., Hoadley A. (2023). Effect of Lithium Ion on the Separation of Electrode Materials in Spent Lithium Ion Batteries Using Froth Flotation. Sep. Purif. Technol..

[69] Robben C., Condori P., Pinto A., Machaca R., Takala A. (2020). X-Ray-Transmission Based Ore Sorting at the San Rafael Tin Mine. Miner. Eng..

[70] Ueda T., Koyanaka S., Oki T. (2024). In-Line Sorting System with Battery Detection Capabilities in e-Waste Using Combination of X-Ray Transmission Scanning and Deep Learning. Resour. Conserv. Recycl..

[71] Wu J., Sun Z., Li D., He W., Yang D., Wu Z., Geng X., Yang H., Wang H., Hu L. (2025). Efficient Estimating and Clustering Lithium-Ion Batteries with a Deep-Learning Approach. Commun. Eng..

[72] Zhu Q., Sun G., Wang P., Sui X., Liu C., Wang J., Zhou J., Wang Z. (2024). Imaging the Space-Resolved Chemical Heterogeneity of Degraded Graphite Anode through Scanning Transmission X-Ray Microscope. J. Power Sources.

[73] Tripathy D.P., Guru Raghavendra Reddy K. (2017). Novel Methods for Separation of Gangue from Limestone and Coal Using Multispectral and Joint Color-Texture Features. J. Inst. Eng. India Ser. D..

[74] Okada N., Nozaki H., Nakamura S., Manjate E.P.A., Gebretsadik A., Ohtomo Y., Arima T., Kawamura Y. (2024). Optimizing Multi-Spectral Ore Sorting Incorporating Wavelength Selection Utilizing Neighborhood Component Analysis for Effective Arsenic Mineral Detection. Sci. Rep..

[75] Neubert K., Wotruba H. (2017). Investigations on the Detectability of Rare-Earth Minerals Using Dual-Energy X-Ray Transmission Sorting. J. Sustain. Metall..

[76] Bischoff P., Kaas A., Schuster C., Härtling T., Peuker U. (2023). Fast and Efficient Evaluation of the Mass Composition of Shredded Electrodes from Lithium-Ion Batteries Using 2D Imaging. J. Imaging.

[77] Korde V.B., Khelkar A.B., Khot S., Malavadakar P., Deshmukh P., Amalraj S. (2026). Advancements of Lithium-Ion Battery Recycling: Transitioning from Traditional Methods to AI and Machine Learning Techniques. Renew. Sustain. Energy Rev..

[78] Rajeev P.A., Dharewa V., Lakshmi D., Vishnuvarthanan G., Giri J., Sathish T., Alrashoud M. (2025). Advancing E-Waste Classification with Customizable YOLO Based Deep Learning Models. Sci. Rep..

[79] Antony Jose S., Cook C.A.D., Palacios J., Seo H., Torres Ramirez C.E., Wu J., Menezes P.L. (2024). Recent Advancements in Artificial Intelligence in Battery Recycling. Batteries.

[80] Zorn M., Ionescu C., Klohs D., Zähl K., Kisseler N., Daldrup A., Hams S., Zheng Y., Offermanns C., Flamme S. (2022). An Approach for Automated Disassembly of Lithium-Ion Battery Packs and High-Quality Recycling Using Computer Vision, Labeling, and Material Characterization. Recycling.

[81] Zhou H., Yu Y., Wang K., Hu Y. (2024). A YOLOv8-Based Approach for Real-Time Lithium-Ion Battery Electrode Defect Detection with High Accuracy. Electronics.

[82] Park K., Choi B. (2018). Requirement of High Lithium Content in Ni-Rich Layered Oxide Material for Li Ion Batteries. J. Alloys Compd..

[83] Anticoi H., Garcia L., Dan G., Sola P., Dorado A.D. (2025). Methodology for Evaluation of Lithium-Ion Black-Mass Battery Recycling. J. Mater. Cycles Waste Manag..

[84] Mistry F., Prasetyo J. (2025). The RoBatt: Battery Sorting Robot Using Epson VT6 and YOLO v8: An Innovative Battery Sorting System That Integrates YOLOv8 Object Detection with an Epson VT6 Robot and Electromagnetic Gripper. J. Appl. Sci. Adv. Eng..

[85] Adewuyi S.O., Anani A., Luxbacher K., Ndlovu S. (2025). From Single-Sensor Constraints to Multisensor Integration: Advancing Sustainable Complex Ore Sorting. Minerals.

[86] Bai Z., Gao P., Chu M., Han Y., Yuan S., Tang J., Li Y., Shi Q., Qiao J., He J. (2025). Artificial Intelligence of Mineral Processing Process: A Review of Research Progress. J. Environ. Chem. Eng..

[87] Qiu J., Zhang Y., Fu C., Yang Y., Ye Y., Wang R., Tang B. (2024). Study on Photofluorescent Uranium Ore Sorting Based on Deep Learning. Miner. Eng..

